# Sulfur and oxygen isotope insights into sulfur cycling in shallow-sea hydrothermal vents, Milos, Greece

**DOI:** 10.1186/s12932-014-0012-y

**Published:** 2014-08-12

**Authors:** William P Gilhooly, David A Fike, Gregory K Druschel, Fotios-Christos A Kafantaris, Roy E Price, Jan P Amend

**Affiliations:** 1 Department of Earth Sciences, Indiana University-Purdue University Indianapolis, Indianapolis, IN, USA; 2 Department of Earth and Planetary Sciences, Washington University in St. Louis, St. Louis, MO, USA; 3 Department of Earth Sciences, University of Southern California, Los Angeles, CA, USA; 4 SUNY Stony Brook, School of Marine and Atmospheric Sciences, Stony Brook, NY, USA; 5 Department of Biological Sciences, University of Southern California, Los Angeles, USA

**Keywords:** Palaeochori Bay, Milos Island, Shallow-sea hydrothermal vents, Phase separation, Sulfur isotopes, Sulfate oxygen isotopes, Anhydrite, Sulfide oxidation

## Abstract

Shallow-sea (5 m depth) hydrothermal venting off Milos Island provides an ideal opportunity to target transitions between igneous abiogenic sulfide inputs and biogenic sulfide production during microbial sulfate reduction. Seafloor vent features include large (>1 m^2^) white patches containing hydrothermal minerals (elemental sulfur and orange/yellow patches of arsenic-sulfides) and cells of sulfur oxidizing and reducing microorganisms. Sulfide-sensitive film deployed in the vent and non-vent sediments captured strong geochemical spatial patterns that varied from advective to diffusive sulfide transport from the subsurface. Despite clear visual evidence for the close association of vent organisms and hydrothermalism, the sulfur and oxygen isotope composition of pore fluids did not permit delineation of a biotic signal separate from an abiotic signal. Hydrogen sulfide (H_2_S) in the free gas had uniform δ^34^S values (2.5 ± 0.28‰, n = 4) that were nearly identical to pore water H_2_S (2.7 ± 0.36‰, n = 21). In pore water sulfate, there were no paired increases in δ^34^S_SO4_ and δ^18^O_SO4_ as expected of microbial sulfate reduction. Instead, pore water δ^34^S_SO4_ values decreased (from approximately 21‰ to 17‰) as temperature increased (up to 97.4°C) across each hydrothermal feature. We interpret the inverse relationship between temperature and δ^34^S_SO4_ as a mixing process between oxic seawater and ^34^S-depleted hydrothermal inputs that are oxidized during seawater entrainment. An isotope mass balance model suggests secondary sulfate from sulfide oxidation provides at least 15% of the bulk sulfate pool. Coincident with this trend in δ^34^S_SO4_, the oxygen isotope composition of sulfate tended to be ^18^O-enriched in low pH (<5), high temperature (>75°C) pore waters. The shift toward high δ^18^O_SO4_ is consistent with equilibrium isotope exchange under acidic and high temperature conditions. The source of H_2_S contained in hydrothermal fluids could not be determined with the present dataset; however, the end-member δ^34^S value of H_2_S discharged to the seafloor is consistent with equilibrium isotope exchange with subsurface anhydrite veins at a temperature of ~300°C. Any biological sulfur cycling within these hydrothermal systems is masked by abiotic chemical reactions driven by mixing between low-sulfate, H_2_S-rich hydrothermal fluids and oxic, sulfate-rich seawater.

## Introduction

Sulfur is critical to the functioning of all living organisms, including energy transduction, enzyme catalysis, and protein synthesis [1]. The sulfur biogeochemical cycle, with its broad range in valence (−2 to +6), exhibits a complex interplay between biotic and abiotic processes in hydrothermal vent ecology. Perhaps the most important biological pathway for H_2_S production in sediment-hosted marine environments is microbial sulfate reduction coupled to organic matter mineralization [2],[3]. Abiotic sources of sulfur in seafloor hydrothermal systems include volcanic inputs (H_2_S and SO_2_) and seawater sulfate that has undergone thermochemical reduction, anhydrite precipitation, or water-rock interactions [4]–[6]. Hydrologic circulation of seawater through cracks and fissures of hot ocean crust results in a net removal of seawater sulfate through the formation of anhydrite (CaSO_4_) [7],[8]. Overall, the exchange between seawater and ocean crust results in significant sources (e.g. Ca and Fe) and sinks (e.g. Mg and S) of elements to the global oceans [8]–[10]. These elemental budgets are primarily derived from investigations of altered basalt in trenches and deep-sea hydrothermal vents in spreading crust (mid-ocean and back-arc spreading centers) [11].

Sulfur (δ^34^S) and oxygen (δ^18^O) isotopes have provided valuable insight into deep-sea hydrothermal processes. The isotopic composition of hydrothermal fluids depends on the relative contributions of different sulfur (or oxygen) sources, their isotopic composition, and any fractionation effect that may occur during rate-limiting chemical or biological reactions. Assuming a simple two end-member system, sulfur within the ocean crust (δ^34^S ≈ 0‰) [12]–[15] can be distinguished from seawater sulfate (δ^34^S_SO4_ = 21.1‰) [16]. However, multiple investigations have shown that the isotopic signature of igneous sulfur is not uniform and that it depends upon oxygen fugacity of the melt [15], extent of melting [17], and water-rock interaction during assent of hydrothermal fluids. Although the subsurface variability can be due to multiple abiotic reactions, direct measurements of xenoliths provides some constraint on the sulfur isotopic composition of the mantle (δ^34^S = −5 to 9‰) [17],[18], and compilations of vent fluids and seafloor sulfide minerals (δ^34^S = −1 to 14‰) as reviewed in [13] approximate these mantle values. In contrast to the slightly ^34^S-enriched igneous contributions, sulfur inputs that have cycled through microbial sulfate reduction are characteristically depleted in ^34^S (Δ^34^S_SO4-H2S_ up to 66‰) [19],[20]. Such low δ^34^S values have been essential in recognizing microbial activity in the deep biosphere within altered marine crust [12]–[14],[21]. Likewise, low δ^34^S (<< 0‰) of hydrothermal seafloor deposits is diagnostic of biogenic H_2_S recycled into the crust during basin-scale subduction of marine sediments [22],[23].

The oxygen isotope composition of global seawater (δ^18^O_H2O_ = 0‰) tends to become ^18^O-enriched during thermal alteration [24]. Isotopic exchange between sulfate oxygen (δ^18^O_SO4_ = 8.7‰) and water is exceptionally slow (10^7^ years) at normal seawater conditions (temperature = 4°C and pH = 8) [25]; however, δ^18^O_SO4_ of residual sulfate increases during microbial sulfate reduction and equilibrium isotope exchange that proceeds through the sulfur intermediate species sulfite [26],[27]. These sulfur and oxygen end-members have been informative in differentiating the relative contributions of seawater and igneous sources to high temperature fluids.

While stable isotope investigations of deep-sea hydrothermal systems (>1600 m water depth) have garnered much attention [12]–[14],[21],[28],[29], their shallow-sea analogs have been largely overlooked [30]–[32]. Volcanic arcs often produce shallow-sea vent systems, and their geochemical cycles can differ demonstrably from those found in mid-ocean ridges. Compared to deep-sea hydrothermal systems, the shallow-sea varieties are generally cooler (<150°C), are under lower hydrostatic pressure (<21.1 bar by definition), and can be found within the photic zone near shore [33],[34]. Several processes can affect the overall composition of discharging hydrothermal fluids. Phase separation is a ubiquitous process in both deep- and shallow-sea hydrothermal systems [11],[35]–[38]. At the low pressures encountered in shallow-sea systems, phase separation often occurs below the critical point of seawater and can be equated to “subcritical” boiling [37],[39]. Thus, vent fluid salinities can vary drastically, from less than 6% up to 200% of normal seawater [11]. This process of phase separation is common to arc-systems and results in wide ranges of major element compositions and base metal precipitation [38],[39]. Water-rock interactions, magma composition and volatile inputs are also highly variable compared to the more uniform basaltic crust of deep-sea systems [38]. While hydrothermal venting can occur along higher permeability fracture zones in both deep- and shallow-sea environments [40], in the latter, these highly advective pathways can become diffusive by passage through overlying sediment [39]–[44]. Shallow-sea sediments have their own recirculation and fluid hydrodynamics that are influenced by wave action, currents, and sediment remobilization [45]–[47]. Furthermore, the discharging fluids in shallow-sea systems need not originate from seawater, but in many cases can be derived from meteoric fluids [39],[48],[49].

Each of these processes, as well as the complex interaction of reduced hydrothermal fluids with oxic seawater, can affect the isotopic composition of dissolved sulfate and H_2_S. One such critical process includes sulfide oxidation mediated by chemical or biological reactions. Much of the H_2_S generated during microbial sulfate reduction or submarine hydrothermal activity is ultimately oxidized back to sulfate through aerobic or anaerobic reactions; however, this eight electron transfer does not proceed in a single step [50]. A variety of intermediate sulfur species (including sulfite, thiosulfate, elemental sulfur, polythionates, and polysulfides) are produced during sulfide oxidation under oxic and anoxic conditions [51]. Once elemental sulfur is present, it can then react with sulfite and H_2_S to form thiosulfate and polysulfides [51]. Although transient and generally short lived, polysulfides and H_2_S are involved in pyrite formation, organic matter sulfurization, and trace metal immobilization [51],[52]. Sulfide oxidation pathways within shallow-sea hydrothermal vents include chemical oxidation and biologically mediated oxidation through chemolithotrophy and phototrophy. Sulfide oxidation with molecular oxygen (O_2_) is relatively slow in the absence of metal catalysts or microorganisms [50],[53] with the abiotic rate of that process in seawater primarily a function of oxygen concentration. The following rate law [54] can represent this reaction:

(1) $$d\;\left[H_{2}S\right]\;/\;\mathrm{dt}=k\;\left[O_{2}\right]\;\left[H_{2}S\right]$$

(2) $$\log\;k=\;10.5\;+\;0.16\mathrm{pH}\;–\;3x10^{3}/T\;+\;0.49\;I^{1/2}$$

where, *k* is in kg of H_2_O mol^−1^ h^−1^[53],[55]. At conditions representative of shallow-sea hydrothermal venting e.g., [39], the half-life of H_2_S at pH 4, 100°C, 0.001 activity H_2_S at an ionic strength (I) of 0.7 would be approximately 8 days (207 hours). In part because the abiotic oxidation kinetics are slow, chemolithotrophic microorganisms can gain energy by catalyzing this process. Aerobic chemolithotrophs can increase the net sulfide oxidation rate by many orders of magnitude, depending on cell density [56].

Environments with biological activity in close proximity to hydrothermal inputs, such as those found in shallow-sea vents, might offer a unique opportunity to explore the relative contributions of biotic and abiotic reactions to local sulfur cycling. The geochemical sulfur transformations in these settings create environmental gradients suitable for microbial activity within marine hydrothermal systems, and the biological utilization imparts additional pathways of sulfur redox chemistry. Here we investigate the impact of shallow-sea hydrothermal activity on marine sulfur cycling as revealed in sulfur and oxygen isotopes of vent fluids in coastal waters of Milos Island, Greece, with the goal of determining whether biological isotopic signals can be detected and distinguished from hydrothermal abiotic reactions. A novel film method was used to document high-resolution (mm-scale) changes in H_2_S abundance in order to best approximate spatial scales relevant to microorganisms [57],[58]. This work further contributes to improved understanding of sulfur and oxygen isotope systematics in shallow-sea hydrothermal systems and associated interactions with the ocean, with important consequences for refining global biogeochemical budgets [59],[60] and for providing modern analogs for evolving chemistry of the ancient ocean [61],[62].

### Site description

**Figure 1:**
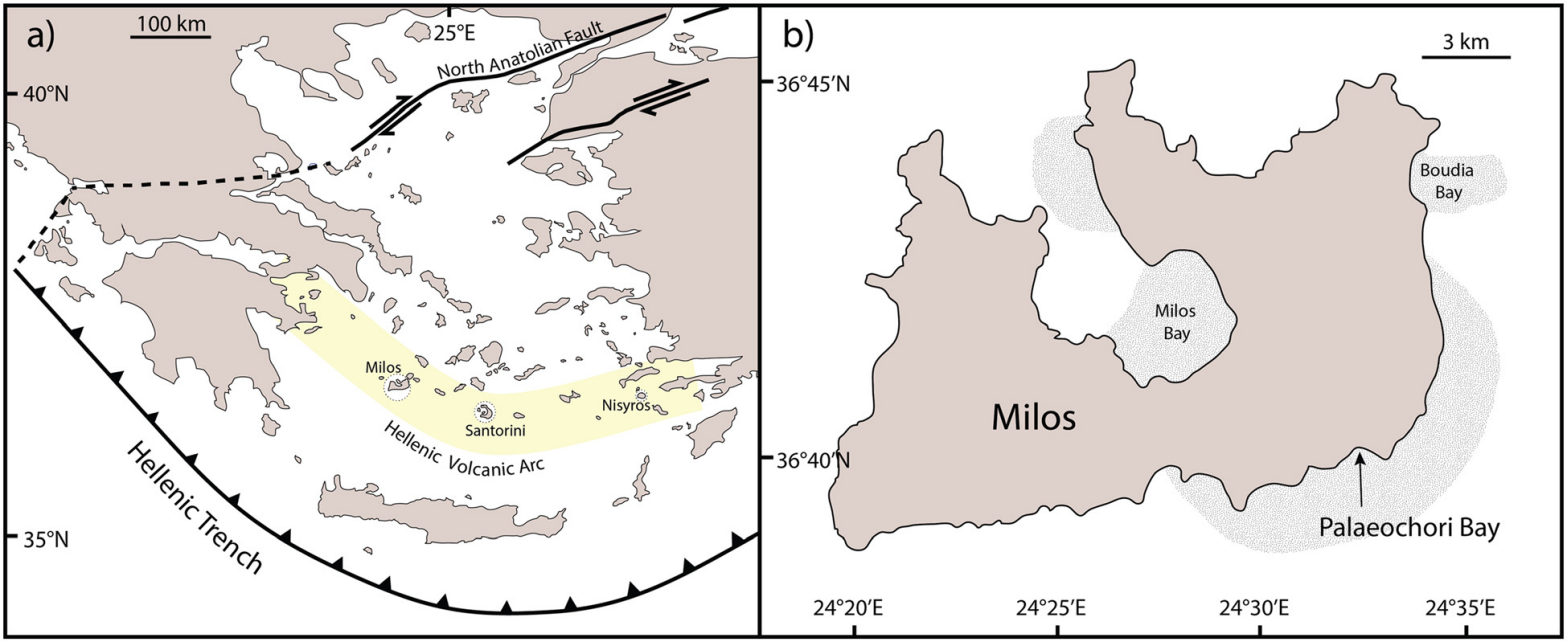
**Map of shallow sea hydrothermal vents. (a)** The hydrothermally active islands of Milos, Santorini, and Nisyros are located along the Hellenic Volcanic Arc. **(b)** Samples were collected in 2011 from shallow (~5 m water depth) submarine hydrothermal sites in Palaeochori Bay. The shaded areas represent the areal extent of hydrothermal activity (34 km^2^) observed around Milos [73]. Figures modified from previous Milos studies [73],[74].

**Figure 2:**
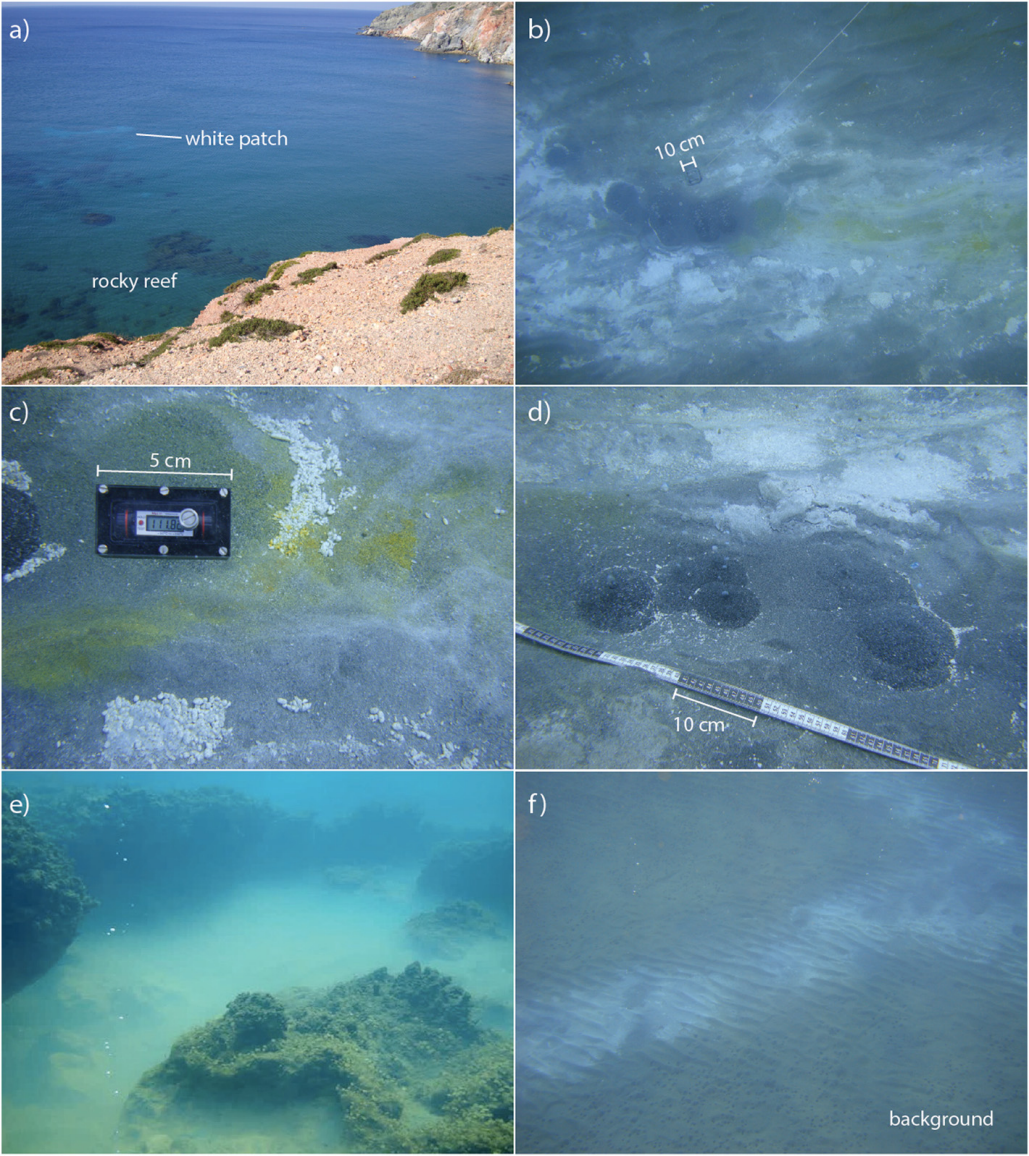
**Pictures of hydrothermal features in Palaeochori Bay. (a)** White patches and a rocky reef as seen from cliffs overlooking the bay. **(b)** The physical appearance of the hydrothermal features was highly heterogeneous, ranging from patches of white to orange/yellow precipitates grading into non-pigmented background sediments. **(c)** The yellow and orange precipitates tended to form toward the center of the features where pore water temperatures were highest. **(d)** Gas flow from several vent mounds formed streams of bubbles in the water column. **(e)** Saline brine accumulated within a depression of a rocky reef. **(f)** Background sediments with no visible evidence for venting or microbial cover were used as control sites.

Milos is an island arc volcano located along the Hellenic Volcanic Arc in the Aegean Sea (Figure 1a). The arc system was formed by convergence between the African and the Aegean continental plates during the closure of the Tethys ocean [63]. Ocean crust subduction and crustal thinning of the continental margin results in magmas of intermediate to felsic composition (andesite, dacite, rhyolite) [64]. Since the last eruption ~90 kya, remnant heat from the dormant system drives hydrothermal circulation in many places on land and in the shallow sea, particularly near the southeastern part of the island (Figure 1b; shaded areas). Hydrothermal venting is manifested as extensive areas of free gas discharge and diffusively venting geothermal fluids along the shoreline to depths of at least 110 m [42],[65],[66] (Figure 1b). The interaction between reduced, H_2_S-rich, hot (up to 111°C), slightly acidic (pH 4–5) hydrothermal fluids and cooler, oxygen-rich, slightly alkaline seawater produces mineral precipitates inhabited by microbial communities on the seafloor that are visible from the shore (Figure 2a). The hydrothermal fluids are highly enriched in H_2_S (up to several millimolar) [39],[41],[42], and elemental sulfur and arsenic-sulfide are common precipitates [39]. The white fluffy coatings (Figure 2b) were approximately 1-cm thick and host chemolithotrophic sulfide oxidizing and sulfate reducing bacteria [41],[67]–[70]. Vent gases emitted in Palaeochori Bay consist predominantly of carbon dioxide (~95% CO_2_), but also contain high concentrations of H_2_S and other volatiles (e.g., CH_4_ and H_2_) [41]. The efflux of fluids charged with carbon dioxide and H_2_S likely support sulfide oxidizing bacteria that inhabit the white regions. However, there appears to be a dynamic sulfur cycle at the site, featuring both biotic and abiotic sulfur oxidation and reduction [67],[68],[70]–[72].

The seabed near our sampling area is composed of rocky reef within meters of the shoreline and sandy sediment throughout the bay (Figure 2a). The entire study area is above wave base (depth of ~ 5 m) and thus is often exposed to wind-driven mixing. The bottom topography includes wave ripples covered with white patches (Figure 2b), yellow and orange precipitates (Figure 2c), rounded mounds of dark, fluidized sand emitting free gas (Figure 2d), and a hyper-saline brine accumulated within the depression of a rocky reef (Figure 2e). The white patches and orange/yellow precipitates are much warmer (>40°C) than the surrounding sediments [39],[68],[75]. Thermal fluids often contained elevated concentrations of Na, Ca, K, Cl, SiO_2_, Fe, and Mn relative to mean seawater concentration [39],[42],[65],[66],[69] and were typically depleted in SO_4_ and Mg [39],[42],[66],[69]. However, recently a low-Cl fluid (depleted by as much as 66% relative to seawater) also depleted in Na, Mg, SO_4_, and Br, was sampled within a few meters of the high-Cl vents [39]. Brown (background) sediments outside of the hydrothermal features have temperatures and pore water chemistry that are similar to ambient seawater e.g., [39] (Figure 2f).

## Methods

### Fieldwork

**Figure 3:**
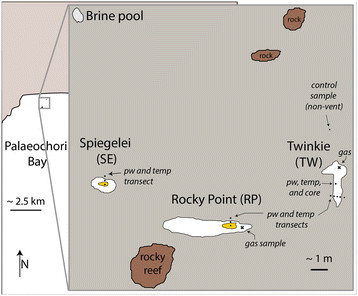
**Location map of the Brine pool, and the white patches of Twinkie (TW), Rocky Point (RP), and Spiegelei (SE).** The center of RP and SE contained large areas of orange precipitate. Pore water samples and temperature measurements were taken along transects within each sampling site. Pore water, temperature and a sediment core were also collected north of the transect in TW. Free gas samples (×) were collected from RP and TW. A station north of TW was collected to provide a control sample from non-vent sediments.

SCUBA divers collected samples (pore waters, water column and free gas) and conducted *in situ* temperature measurements in 2011 at study sites ‘Rocky Point’, ‘Spiegelei’, ‘Twinkie’ and the ‘Brine pool’ (Figure 3). White patches were observed within Rocky Point, Spiegelei, and Twinkie. Orange, interspersed with yellow, precipitates were found in the central areas (approximately 25 to 50 cm in diameter) of Rocky Point and Spiegelei. The patches at Twinkie were predominately white in color, with some small areas of yellow precipitate (approximately 1 cm in diameter) located toward the center of the site. Pore water sampling was conducted along transects that extended from the center of the hydrothermal vents into gray sediment (a distance of approximately 1 m) to provide environmental context between vent and adjacent sediment. Background samples were also collected from a control site located away from actively venting sediments (Figure 3; ‘control sample’). Discrete pore water samples were collected in 5 to 10-cm depth intervals to a maximum depth of 20 cm using a pipette tip attached to tygon tubing and a 60 ml syringe [39]. The first 20 ml were discarded to decrease potential seawater contamination during sampling. Seawater was collected from the bay surface near the shore and away from any apparent venting activity. A second seawater sample was collected from the bottom water overlying the Twinkie study site. A white patch area north of the transect at Twinkie was also cored with polycarbonate tubes that were sealed underwater. The pore water chemistry of the cored sediments was analyzed by voltammetry (*described in 3.2 Analytical*). Surface sediments were also collected from each site using 50 ml centrifuge tubes.

Samples of free gas were collected from active vents through an inverted funnel placed over an area where free gas bubbles streamed through the seafloor sediments (Figure 3; ‘×’). Outflow from the funnel was collected in a syringe with luer lok fittings and sealed with a stopcock. Dissolved H_2_S in filtered pore waters (0.2 μm membrane filters) and free gas was precipitated as ZnS by addition of 3% zinc acetate (wt/v) within 1 hour after completion of the dive. Pore water temperatures were determined with a digital thermometer in an underwater housing.

Pore water pH was measured using a WTW pH meter and a MIC-D electrode with built-in temperature compensation, which had a precision of 0.1 pH units. Dissolved sulfate and chloride concentrations were determined by ion chromatography on a Dionex DX600 with a ED50 detector and KOH eluent gradient. Analytical precision of field and laboratory duplicates had a reproducibility of ±5%.

**Figure 4:**
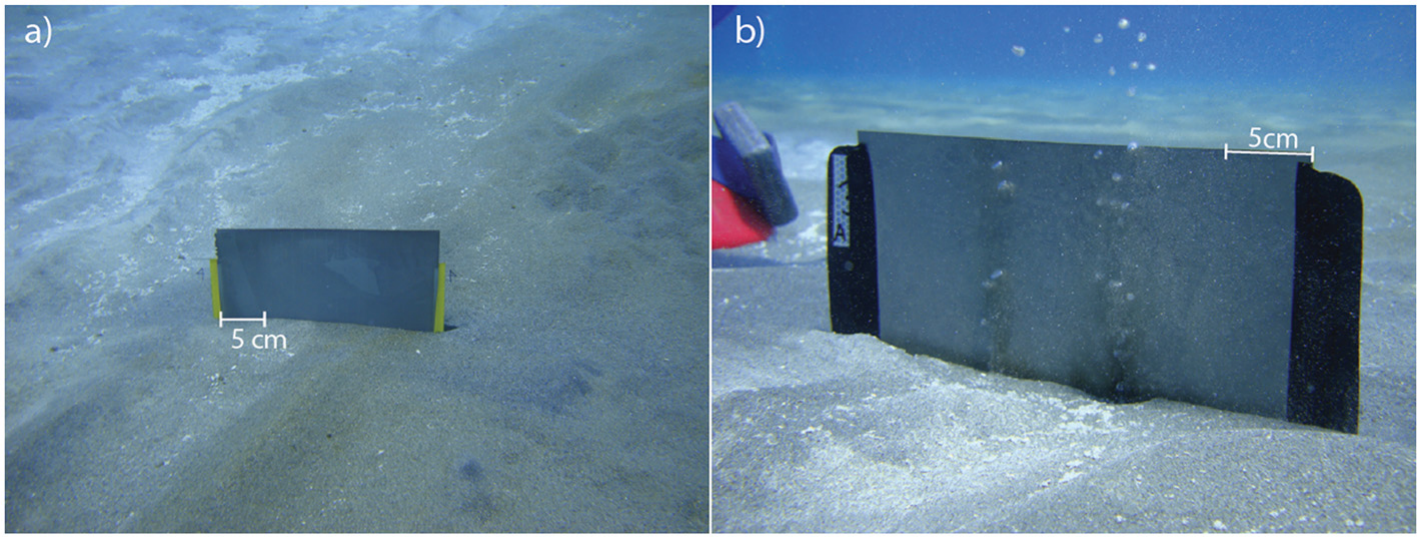
Films were deployed by SCUBA to capture free sulfide (a) dissolved in the pore waters and (b) venting from gas mounds.

**Figure 5:**
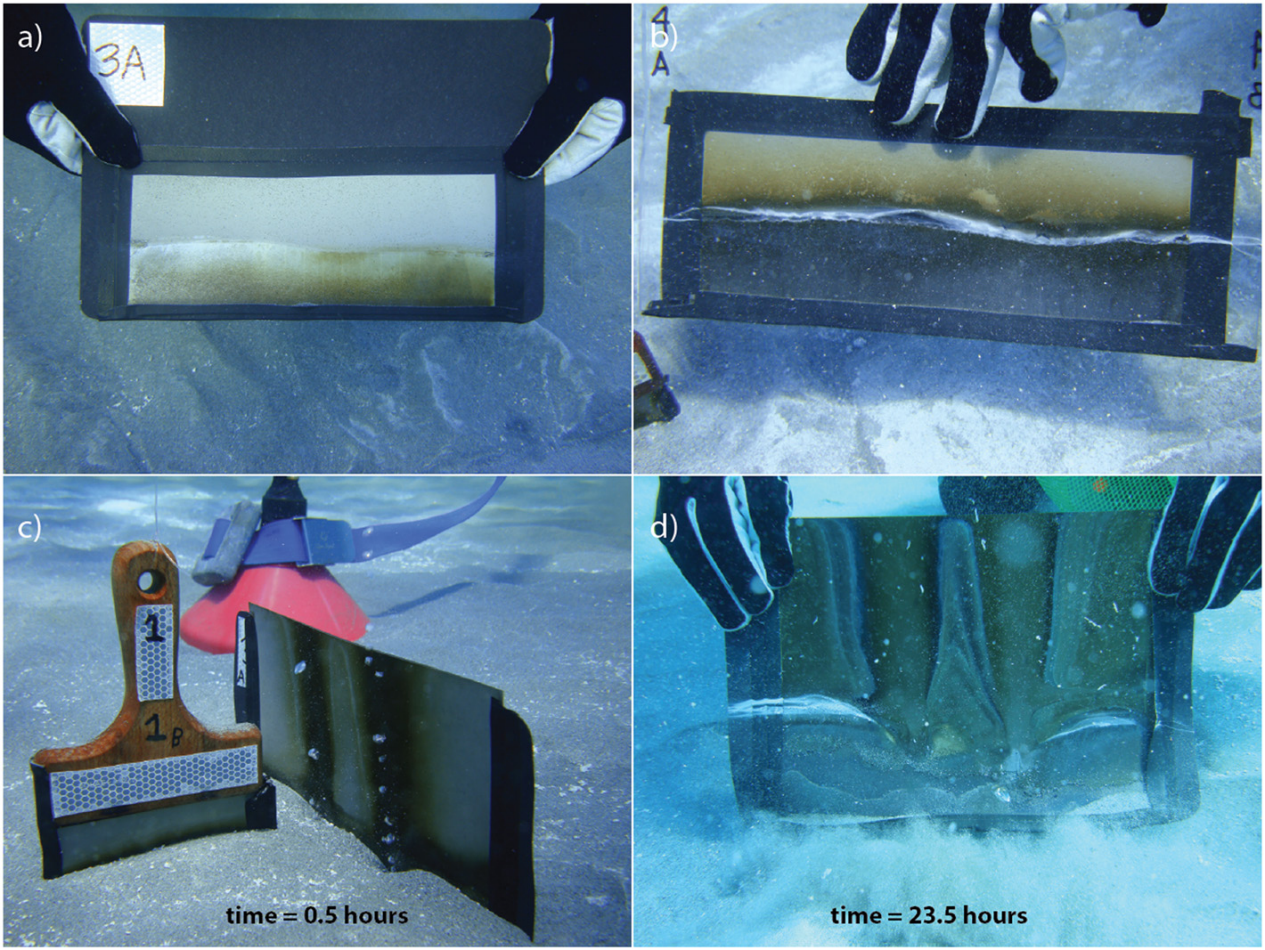
**The film-method captured the highly variable sulfide flux across the hydrothermal sites. (a)** The undulating surface of ripple marks and the position of the sediment water interface were retained on films. **(b)** White filamentous material indicates the position of the sediment and the sulfide-staining above the interface indicates sulfide diffusion directly into the bottom waters. **(c)** Gas plumes imprinted on a film placed near an active vent mound within 30 minutes and **(d)** after 23 hours.

Silver halide-embedded photographic films (Ilford Delta 100) were deployed to precipitate dissolved H_2_S within white- and yellow-stained sediment, and background (non-vent) sediments (Figure 2b, c, d, f). Films were also deployed in diffusively venting (Figure 4a) and actively venting (Figure 4b) sediments. Dissolved H_2_S reacted with the silver in the film surface to form Ag_2_S. The degree of coloration is proportional to the mass of H_2_S that reacted with the film (Figure 5). Dissolved H_2_S that precipitated in the films represents a time-averaged H_2_S flux for sulfur isotope analysis. Films (4 x 5 inches or 8 x 10 inches) were deployed for at least 1 hour and up to 24 hours to ensure quantitative reaction of the silver in the resins with dissolved and free gas H_2_S. The films were stable within the environmental pH range of 4 to 8. Trial deployments revealed that the silver-containing resin separated from the acetate backing of the film when exposed to temperatures above 90°C. Noting this temperature effect, films were successfully used in sediment temperatures up to ~85°C.

### Analytical

Dissolved H_2_S concentrations of syringe-sampled fluids were measured by voltammetry on a DLK-60 potentiostat (Analytical Instrument Systems) using a three electrode system consisting of a 100 μm Au-amalgam working electrode, Ag/AgCl reference electrode, and Pt counter [76]. Voltammetric signals are produced when redox-active dissolved or nanoparticulate species interact with the surface of the Au-amalgam (Au-Hg alloy) working electrode. Electron flow, resulting from redox half-reactions occurring at specific potentials at the 100 μm Au-amalgam diameter working electrode surface, is registered as a current that is proportional to concentration [77]–[79]. Cyclic voltammetry was performed between −0.1 and −1.8 V (vs. Ag/AgCl) at a scan rate of 1000 mV s^−1^ with a 2 s conditioning step. Aqueous and nanoparticulate sulfur species that are electroactive at the Au-amalgam electrode surface of direct relevance to this study include HS^−^, H_2_S, S_8_, polysulfides, S_2_O_3_^2−^, HSO_3_^−^, and S_4_O_6_^2−^[80],[81]. Calibrations were performed in seawater collected on site utilizing the pilot ion method [82],[83]. Precision of the data using this technique is typically within 2-3% at these sulfide levels (<1000 μM); however, uncertainties tied to inter-electrode variability and compound analytical error associated with utilizing Mn^2+^ for calibration in the field can yield overall analytical uncertainties up to 10% [82],[83].

The δ^34^S values of the preserved sulfur compounds were measured with an isotope ratio mass spectrometer (Thermo Delta V Plus, at WUSTL) coupled under continuous flow to an elemental analyzer (Costech Analytical ECS 4010). Dissolved sulfate splits were precipitated as BaSO_4_ by addition of saturated barium chloride solution. H_2_S fixed as ZnS was reprecipitated as Ag_2_S by addition of silver nitrate solution. H_2_S trapped on photographic films were liberated by chromium reduction [84] and precipitated as Ag_2_S. Samples were mixed with vanadium pentoxide to ensure complete combustion. The oxygen isotope composition of sulfate (δ^18^O_SO4_) was measured by pyrolysis (Thermo TC/EA) and gas source mass spectrometry (Thermo Delta V Plus, at IUPUI). Graphite was added to each sample to promote consistent pyrolysis. The oxygen or sulfur isotope composition (^*x*^E = ^18^O or ^34^S) was reported in per mil (‰) according to the equation:

(3) $$\delta^{X}\;E=\left(\frac{R_{\mathrm{sample}}}{R_{\mathrm{standard}}}-1\right)\times 1000,$$

where the isotopic ratio (R = ^18^O/^16^O or ^34^S/^32^S) of the sample is normalized to the isotopic ratio of the international standard for Vienna Standard Mean Ocean Water (VSMOW) or Vienna Canyon Diablo Troilite (VCDT), respectively. Oxygen isotope reference materials included IAEA-SO6 (δ^18^O = −11.0‰), NBS-127 (δ^18^O = 8.7‰), and IAEA-SO5 (δ^18^O = 12.0‰). Sulfur isotope values were calibrated against international reference materials IAEA-S3 (δ^34^S = −32.55‰), IAEA-S1 (δ^34^S = −0.3‰), and NBS-127 (δ^34^S = 21.1‰). For both oxygen and sulfur, linear regression was used to correct unknowns to the international reference values and to account for scale compression. Analytical precision for standards and replicate samples was ±0.2‰ (1σ) for oxygen and ±0.3‰ (1σ) for sulfur isotopes.

Sediments were analyzed for their mineral content by a combination of optical microscopy, X-ray Diffraction (XRD), and Raman microscopy. Thin sections of 3 representative areas where prepared by Vancouver Petrographics and analyzed together with grain mounts using an Olympus BX-53 microscope, a DeltaNu Rockhound portable Raman spectrometer with microscope attachment, and a Siemens D5000 XRD. Surface samples from background, white and yellow sediments were analyzed for total organic carbon (TOC) concentration. The sediments were decarbonated with 1 N HCl, rinsed three times with deionized water, and dried. Organic carbon content was measured on an elemental analyzer (Costech Analytical ECS 4010).

## Results

The films method captured the spatial and temporal distribution of H_2_S within the upper few cm of sediment (Figures 4 and 5). Sedimentary structures, such as crests and troughs of wave ripples, as well as the position of the sediment-water interface were preserved on the film (Figure 5a). Film images also retained evidence for H_2_S efflux from the pore waters into the bottom water (Figure 5b) and the extent of the hydrothermal plume (Figures 5c and d). These transient and highly dynamic features of surficial venting preserved by the films are not readily sampled by static pore water extractions (e.g., rhizons, squeezing, or centrifugation) or by water column collections (e.g., Niskin or *in situ* pumping).

**Figure 6:**
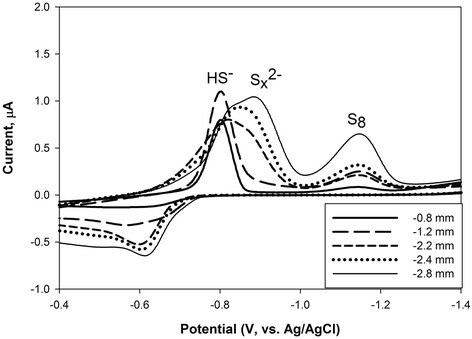
**Representative voltammetric scans from pore waters through a core collected from Twinkie, showing sulfide-dominated conditions and conditions with higher elemental sulfur corresponding to higher levels of polysulfide.** Sulfide sourced from thermal fluids oxidizes to elemental sulfur, additional sulfide then reacts with this elemental sulfur to form polysulfide, a key part of sulfur intermediate chemistry influencing overall sulfur cycling in this system.

Voltammetric analysis of pore water sulfur speciation showed significant influence of precipitated elemental sulfur on the dissolved sulfur speciation present. Voltammetric scans (Figure 6) through a few millimeters of the upper portion of the core collected from Twinkie indicate the presence of micromolar levels of polysulfides when electroactive elemental sulfur is present. In contrast, there is no measurable polysulfide when voltammetric scans indicate low levels of elemental sulfur. This association with elemental sulfur, H_2_S, and polysulfide were observed in both core samples and in the syringe-sampled pore water. Based on equilibrium thermodynamics [85], this association, summarized by the reaction:

(4) $$\left(n,-,1\right)/8S_{8}\left(s\right)+HS^{-}⇆\;{S_{n}}^{2-}+\;H^{+}$$

should yield a relatively low level of polysulfide (S_*n*_^2-^) at pH between 4 and 8 (the range of observed pH in the system). Calculated total polysulfide levels [85] would be 55 nM at pH 4 and 20 μM at pH 8. However, our scans indicate a much more significant yield (at the pH for the scans in Figure 6 one would expect sub-micromolar total polysulfide concentrations, but the signal is a magnitude closer to one hundred micromolar polysulfide concentration). Samples without measureable elemental sulfur did not indicate the presence of measureable polysulfide. The observation of polysulfide, at concentrations higher than calculated equilibrium values, suggests the polysulfide levels seen in these core pore waters may be affected by other reactions than simply reaction (4).

Temperature and chemical compositions (including isotope values) of seawater, brine, and free gas are given in Table 1. Temperatures were elevated in the brine pool (46.8°C) and the free gas (>75°C at sites of venting) compared to seawater (22.1°C). The brine pool had high chloride concentrations (911.7 mM) and low sulfate (19.9 mM) relative to local seawater ([Cl] = 620.3 mM; [SO_4_] = 32.5 mM). The isotopic composition of surface seawater in Palaeochori Bay was δ^34^S_SO4_ = 21.2‰ and δ^18^O_SO4_ = 9.0‰. The δ^34^S_H2S_ value of free gas from Twinkie and Rocky Point showed little variability (δ^34^S_H2S_ = 2.5 ± 0.28‰, n = 4) (Table 1). Free gas H_2_S samples were not collected from the Spiegelei site.

**Table 1:** Fluid and free gas chemical data

	**Depth (cm)**	**pH**	**Temp. (°C)**	**Cl (mM)**	**SO**_**4**_**(mM)**	**H**_**2**_**S(μM)**	**δ**^**34**^**S**_**SO4**_**(‰)**	**δ**^**18**^**O**_**SO4**_**(‰)**	**δ**^**34**^**S**_**H2S**_**(‰)**
**Seawater**									
Near shore, surface water	*-*	7.91	22.1	620	32.5	*-*	21.2	9.0	0.0
Twinkie site, bottom water	*-*	*-*	*-*	*-*	*-*	*-*	21.8	9.1	*-*
**Brine pool**									
Brine pool west of beach	*-*	6.30	46.8	912	19.4	0.0	19.9	9.0	*-*
**Free gas**									
Vent area, Twinkie site	*-*	*-*	*-*	*-*	*-*	*-*	*-*	*-*	2.7
	*-*	*-*	*-*	*-*	*-*	*-*	*-*	*-*	2.1
	*-*	*-*	*-*	*-*	*-*	*-*	*-*	*-*	2.8
Rocky Point	*-*	*-*	*-*	*-*	*-*	*-*	*-*	*-*	2.4
**Pore water**									
*Rocky Point (RP)*									
Orange center	10	4.38	88.6	929	8.7	92.0	*-*	*-*	*-*
	20	4.02	92.5	890	7.9	109.0	*-*	*-*	*-*
White area	10	5.32	67.9	745	20.8	257.0	21.2	*-*	2.7
	20	5.33	79.4	731	20.8	246.0	21.9	*-*	2.9
White area	10	5.19	61.3	718	25.5	198.0	20.2	*-*	2.6
	20	5.21	68.0	734	25.9	186.0	20.7	*-*	2.7
Orange/Yellow area	5	4.13	87.5	944	8.3	24.0	17.3	9.0	*-*
	15	4.12	97.4	952	8.8	18.0	17.6	9.1	*-*
*Spiegelei (SE)*									
Orange/Yellow center	5	4.94	79.6	960	22.0	9.0	18.2	9.4	2.9
	15	4.29	90.4	937	17.3	81.0	17.9	9.6	2.0
White outer area	5	5.21	76.5	884	17.6	9.0	20.1	9.3	1.9
	15	5.31	83.3	871	17.2	34.0	20.5	9.5	2.7
Gray fringe	5	5.72	28.3	788	21.6	0.0	21.0	9.7	*-*
	15	5.73	41.4	796	21.9	0.0	21.3	9.7	*-*
*Twinkie (TW), west side of transect*									
Yellow/White center	5	5.34	52.8	561	27.5	253.0	21.3	8.7	2.2
	10	*-*	64.3	*-*	*-*	*-*	*-*	*-*	*-*
	15	5.42	69.6	563	29.9	286.0	20.4	8.9	*-*
	20	*-*	71.5	*-*	*-*	*-*	*-*	*-*	*-*
White area near edge	5	5.26	42.6	620	31.7	231.0	21.7	8.8	2.3
	10	*-*	51.7	*-*	*-*	*-*	*-*	*-*	*-*
	15	5.29	58.4	639	32.9	227.0	21.4	8.8	2.7
	20	*-*	62.7	*-*	*-*	*-*	*-*	*-*	*-*
Gray area near edge	5	5.24	33.5	633	35.3	247.0	21.6	9.1	3.3
	10	*-*	39.9	*-*	*-*	*-*	*-*	*-*	*-*
	15	5.20	45.5	630	32.7	239.0	21.6	9.1	3.2
	20	*-*	49.6	*-*	*-*	*-*	*-*	*-*	*-*
*Twinkie (TW), east side of transect*									
Yellow/White center	5	5.22	48.1	586	29.5	95.0	21.3	8.9	2.8
	15	5.18	67.8	583	29.3	129.0	21.3	8.9	2.8
White area near edge	5	5.22	40.3	604	30.6	82.0	21.6	8.2	2.6
	15	5.22	60.0	613	31.2	134.0	21.5	8.8	3.0
Gray area near edge	5	5.22	30.7	628	32.5	114.0	21.0	8.8	3.1
	15	5.21	46.7	634	33.2	127.0	21.6	9.0	2.6
*Twinkie (TW), north of transect*									
White area	5	4.98	42.3	544	26.5	606.0	21.1	8.6	2.6
	15	4.98	60.5	529	26.2	992.0	21.5	8.6	2.5
*Control (north of TW)*									
Background mud	5	7.45	22.1	638	32.8	6.0	21.2	8.6	*-*
	15	7.36	23.0	640	33.0	42.0	21.0	8.5	*-*

“-” indicates either no data or not applicable.

**Figure 7:**
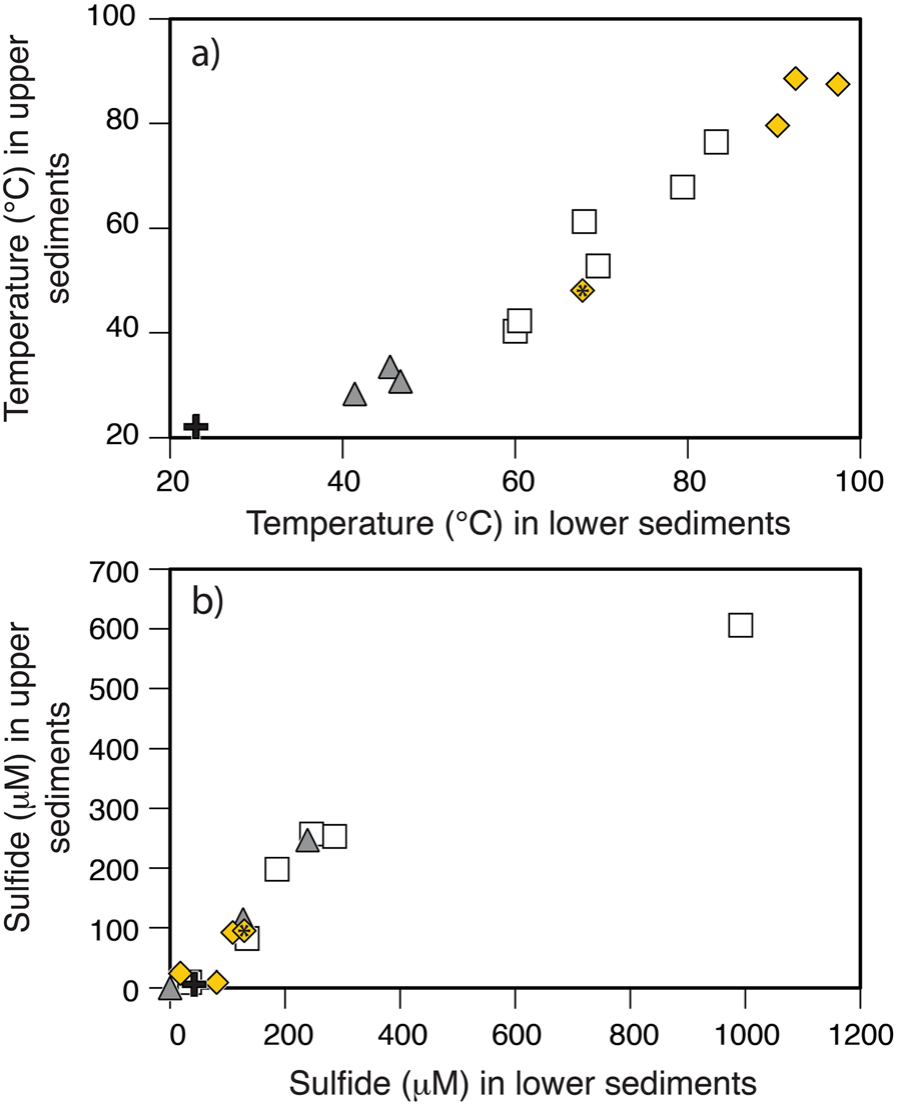
**Changes in temperature and sulfide with depth in background and hydrothermal sediments.** Relative to background sediment distal from venting (+), the **(a)** temperature increases with depth and toward the center of the vent features from gray sediments fringing the hydrothermal sites (▲), to the white patches (□) and orange/yellow precipitates (◆). The precipitates in Rocky Point and Spiegelei were both orange and those in Twinkie (symbol marked with ‘*’) were yellow in color. **(b)** Dissolved H_2_S concentrations also increase with depth in the gray and white sediments but are lowest in the yellow areas. Paired temperature and sulfide measurements in Twinkie and Spiegelei were made at 5 cm (the upper sediments) and 15 cm (lower sediments). Data from Rocky Point were measured at sediment depths of 10 cm and 20 cm.

Pore waters (5–20 cm depth) exhibited a broad range in physical properties and chemistry (Table 1). The hydrothermal sites were moderately acidic (pH 4.0-5.7) and those from background sediments were circumneutral (pH ~ 7.4). Temperature profiles within the hydrothermal sites increased linearly with depth (Figure 7a). The temperatures in the upper 15 cm of background sediments were 22.1 to 23.0°C, and those of the pore waters in white patches were much higher (61.1 ± 13.6°C, n = 14). The temperatures of the yellow precipitate in Twinkie (48.1 to 67.8°C) were similar to the ranges observed in all white patches studied. In contrast, the highest temperatures were measured in orange precipitates of Rocky Point and Spiegelei, which ranged from 79.6 to 97.4°C. Temperatures of gray sediments collected along the margin of the vent areas plot along a gradient between vent influenced sediments (white and orange/yellow) and non-vent sediments (background) (Figure 7a).

**Figure 8:**
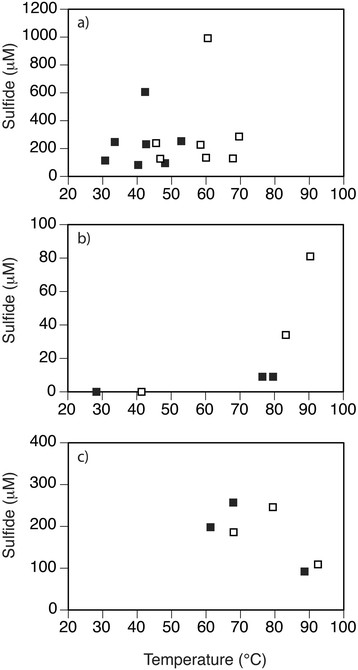
**Sulfide (S) and temperature (T) relationships observed in hydrothermal sites (a) Twinkie, (b) Spiegelei, and (c) Rocky Point.** S/T measurements were made at 5 cm (closed symbols) and 15 cm (open symbols) sediment depth in **(a)** Twinkie and **(b)** Spiegelei, and at 10 cm (closed symbols) and 20 cm (open symbols) in **(c)** Rocky Point.

H_2_S concentrations were higher in areas of seafloor covered in white patches ([H_2_S]_max_ = 992 μM) than either in gray sediments ([H_2_S]_max_ = 247 μM), orange/yellow precipitates ([H_2_S]_max_ = 129 μM), or background (non-vent) sediments ([H_2_S]_max_ = 42 μM) (Figure 7b). The paired H_2_S and temperature data generally increased with depth in Twinkie and Spiegelei (Figures 8a and b). Although there are fewer paired measurements of H_2_S concentrations (S) and temperature (T) in Rocky Point, the S/T relationship decreases within the orange sediments (Figure 8c).

Pore water chloride concentrations in control sediments (~639 mM) were similar to those of ambient seawater (620.3 mM) (Table 1). Chloride levels in Twinkie pore waters (529.3 to 639.2 mM) were less than or equal to that of seawater, and those at Rocky Point and Spiegelei were elevated (731.2 to 959.5 mM), similar to the nearshore brine pool (911.7 mM). In contrast, sulfate concentrations at Rocky Point and Spiegelei (8.7 mM to 25.9 mM) were considerably lower than in seawater, whereas those at Twinkie (26.2 to 35.3 mM) were slightly lower to slightly above local seawater (32.5 mM).

**Figure 9:**
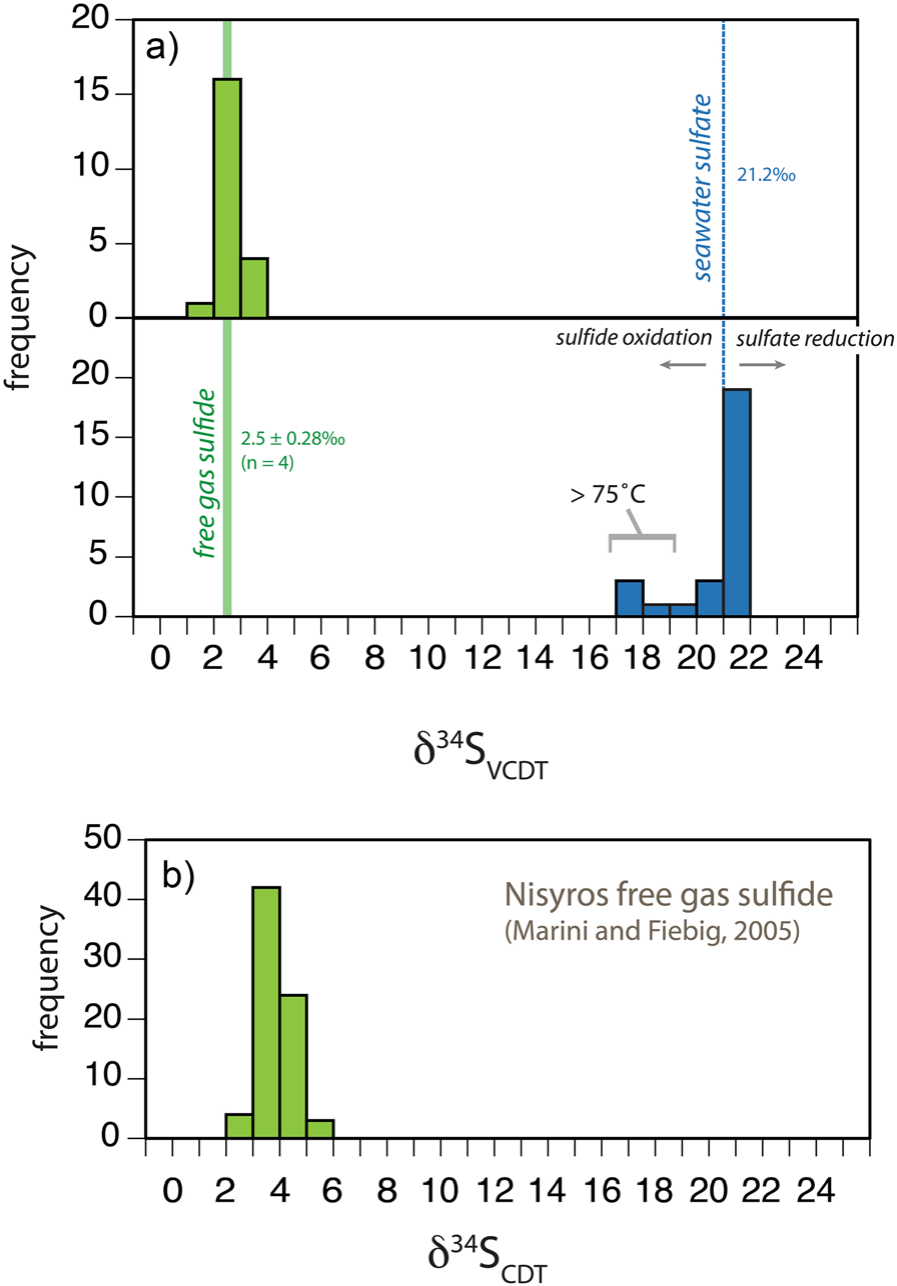
**Sulfur isotope compositions of pore fluids and free gas samples. (a)** Frequency distribution of δ^34^S values for sulfide (green) and sulfate (blue) collected from Palaeochori Bay, Milos (this study). The vertical solid line is the average of free gas sulfide in Palaeochori Bay (δ^34^S = 2.5‰) and the width of the line is the standard deviation of all measurements (±0.28‰, n = 4). The dashed vertical line represents seawater sulfate (δ^34^S = 21.2‰). **(b)** Frequency distribution of free gas sulfide δ^34^S at Nisyros Island [30]. Note, the δ^34^S values for this study **(a)** were normalized to VCDT and the literature values **(b)** were referenced to CDT.

**Figure 10:**
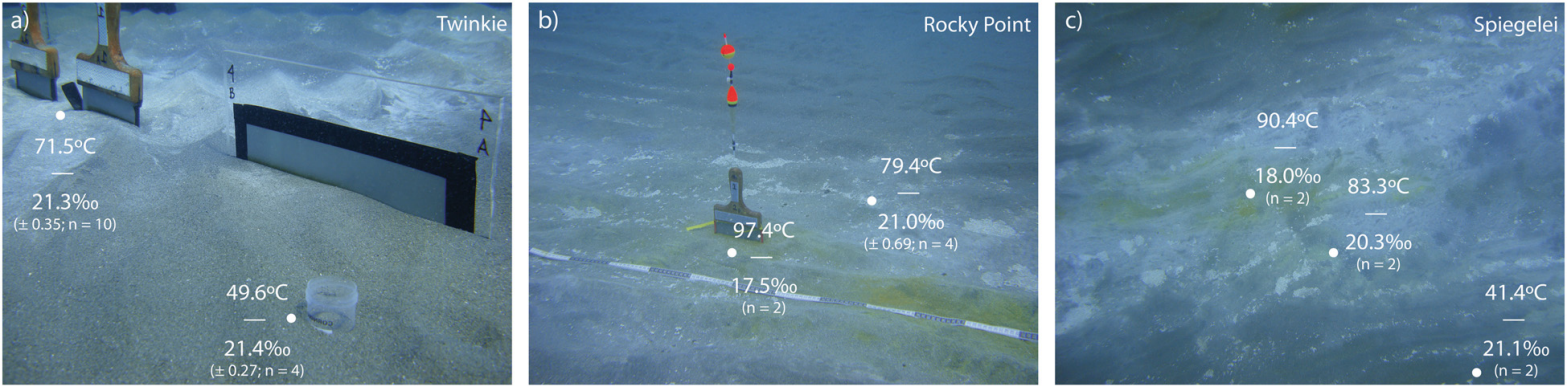
**Maximum temperatures and average pore water δ**^**34**^**S**_**SO4**_**for discrete samples collected 5 to 20 cm below the sediment water interface at hydrothermal sites (a) Twinkie, (b) Rocky Point, and (c) Spiegelei.** The color of the surficial sediments varied from orange/yellow, white, and gray. δ^34^S_SO4_ decreased as temperatures increased toward the center of each feature.

**Figure 11:**
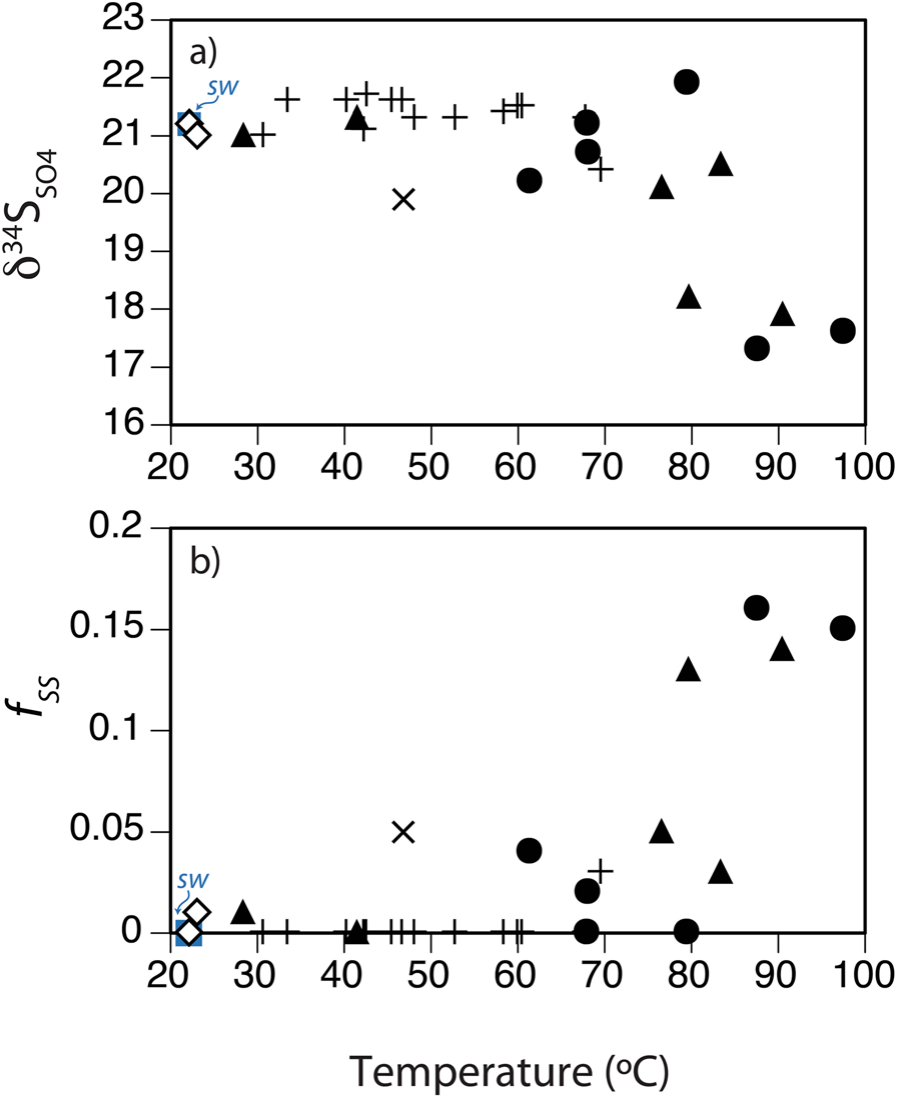
**Relationship between pore water δ**^**34**^**S**_**SO4**_**and temperature. (a)** δ^34^S_SO4_ decreases with increasing temperature across the hydrothermal sites Twinkie (+), Rocky Point (●), and Spiegelei (▲), relative to the Brine pool (×), background sediments (◇), and surface seawater sulfate (sw; ■). **(b)** A mass balance model suggests an increasing fraction of secondary sulfate (*f*_*SS*_) in sites with elevated temperatures.

**Figure 12:**
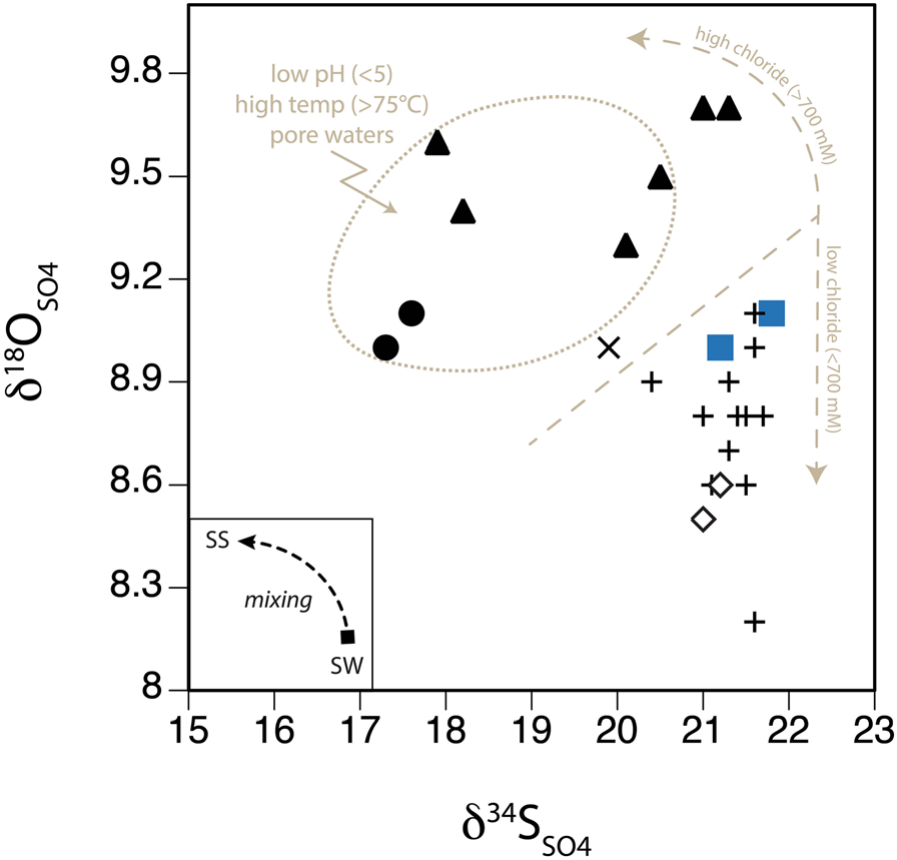
**δ**^**18**^**O**_**SO4**_**and δ**^**34**^**S**_**SO4**_**of pore water in the hydrothermal sites Twinkie (+), Rocky Point (**●**), and Spiegelei (**▲**), relative to the Brine pool (×), background sediments (**◇**), and seawater (**■**).** The inset illustrates the potential mixing trajectory between seawater sulfate (sw) and secondary sulfate (ss).

**Figure 13:**
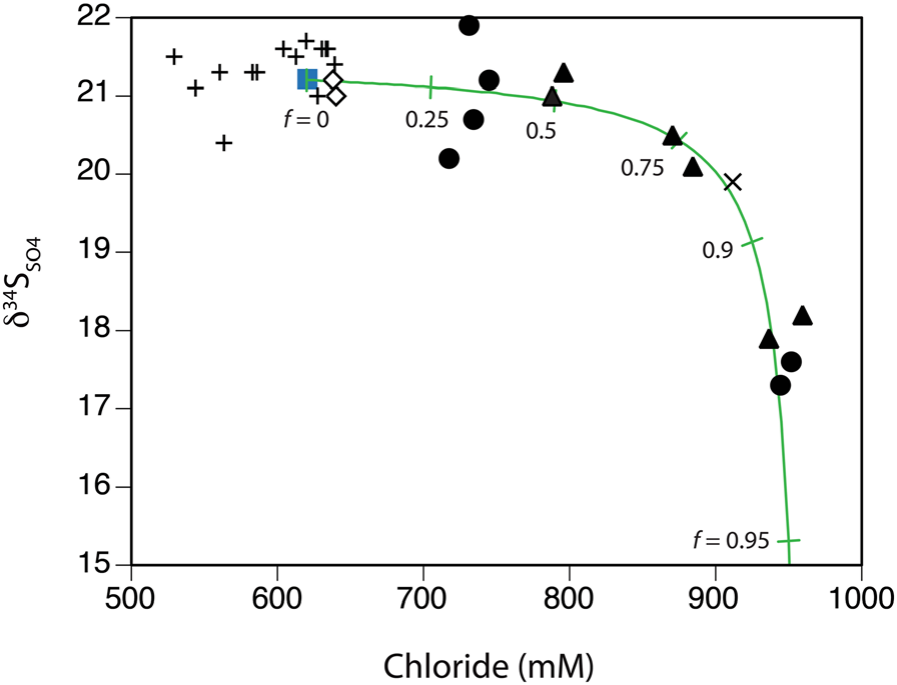
**δ**^**34**^**S**_**SO4**_**values exhibit an inverse relationship with chloride concentrations.** A conservative mixing model demonstrates elevated contributions of high-Cl fluid in Rocky Point (●), Spiegelei (▲), and the Brine pool (×), relative to Twinkie (+), background sediments (◇), and surface seawater (■).

The pore water isotope signatures appear to be overprinted by abiotic chemical reactions. The δ^34^S increase in residual sulfate and associated low δ^34^S in *in situ* H_2_S production expected for microbial sulfate reduction was not observed in pore waters collected in this study. The isotopic composition of pore water H_2_S was constant (δ^34^S_H2S_ = 2.7 ± 0.4‰, n = 21) across all sites and similar to that in vent gas (2.5‰) (Figure 9a). The δ^34^S_SO4_ that was identical to seawater was measured in pore waters sampled from background sediment and from Twinkie (Figure 9a). The exception was a lower δ^34^S_SO4_ value (20.4‰) of a high temperature sample (69.6°C) from Twinkie (Table 1). Pore waters in higher temperature sediments (>75°C) all decreased in δ^34^S_SO4_ (Figure 9a), which is a trend inconsistent with microbial sulfate reduction. When viewed spatially, the maximum temperatures were observed toward the center of each hydrothermal site (Figure 10). Although there is little variation in Twinkie δ^34^S_SO4_ (Figure 10a), Rocky Point and Spiegelei exhibited a pronounced decrease in δ^34^S_SO4_ as temperatures increased (Figures 10b and c). The lowest δ^34^S_SO4_ values (17.3‰ and 17.6‰) within these sites were observed at temperatures above 75°C in the orange zone of Rocky Point. A cross-plot of temperature and δ^34^S_SO4_ further demonstrates the overall trend of low δ^34^S values at high temperatures (Figure 11a). These high temperature, low δ^34^S_SO4_, pore waters also had the highest δ^18^O_SO4_ values (~9.5‰) (Figure 12). The more acidic (pH <5), warmer (>75°C), and more chloride-rich (>700 mM) pore waters of both Rocky Point and Spiegelei were ^18^O-enriched relative to ambient seawater sulfate (δ^18^O_SO4_ = 9.0‰). In contrast, pore waters in Twinkie and background sediments tended to have lower chloride concentrations (<700 mM) and lower δ^18^O_SO4_. Chloride concentrations appear to have a more direct relationship with δ^34^S_SO4_, that decreases as chloride increases (Figure 13). Pore water freshening (chloride concentrations less than seawater) does not appear to influence the δ^34^S_SO4_ in Twinkie (Figure 13; ‘+’).

The sediments collected from each site were screened by microscopy and for their element compositions. Polarized light microscopy (PLM) analyses of thin sections from three separate areas (white sediment, yellow sediment, and background (non-vent) sediments) indicate very similar mineralogical compositions with minor amounts of elemental sulfur as individual grains and as part of a coating. XRD analysis and thin section PLM indicates a predominance of quartz, with modal percentages via each technique estimated at over 90%, with minor clay and feldspar content but no calcite; this is in contrast to a study on sands in a different part of Milos that contained significantly more calcite, clay, and chlorite with much lower quartz content [86]. PLM analysis measured observable elemental sulfur particles of several microns in size, but in quantities <1%. Yellow and white coloration of the grains visible in stereomicroscope images is not visible in thin section, suggesting a very thin coating or reaction of these coatings with the epoxy during sample preparation. Raman spectroscopy of selected grains yields a weak signal for elemental sulfur (normally a strong Raman scatterer), suggesting the visible thin coating of material is at least partly elemental sulfur. The organic carbon concentrations in background, white and yellow sediments were very low (TOC = 0.04 - 0.08%).

## Discussion

### Geochemical variability

We observed chemical and isotopic variability that spanned broad spatial scales. At the scale of the geologic feature of the Hellenic Volcanic Arc (Figure 1a), the δ^34^S values of free gas H_2_S collected from Palaeochori Bay were highly uniform (2.5 ± 0.28‰, n = 4) and similar to fumorolic H_2_S (3.7 ± 0.6‰, n = 73) from Nisyros Island, >300 km away [30]. Consistency between volcanogenic δ^34^S_H2S_ (Figure 9) from these two islands implies a regional control of H_2_S delivery.

At the local scale, chemical variability at vent sites in Palaelochori Bay is best explained by mixing. This is evidenced by changes in seafloor temperature that spanned from that of ambient seawater (22.1°C) to that of hydrothermal inputs (97.4°C). Temperature is often used as a proxy for evaluating the extent to which a hydrothermal fluid mixed with overlying seawater, and the shallow temperature gradients at Spiegelei, Rocky Point and Twinkie imply focused fluid flow at the center of the hydrothermal features (Figure 10). The concentric zonation observed at Rocky Point and Speigelei suggests that seafloor coloration is qualitatively linked to seafloor temperature and associated mineralization, ranging from orange (high temperatures) to white (intermediate temperatures) and gray (lower temperatures) (Figure 7a). The orange precipitates were only found in the warmest regions (>70°C) of the hydrothermal features surveyed in our study area. Small (~1 cm diameter) patches of yellow precipitates interspersed in the white mat were a more common precipitate. The yellow surface manifestations of fluid flow exhibited temperatures that were similar to those measured in the white patches. H_2_S concentrations however, had more direct relationship to temperature (Figure 7). For example, the H_2_S concentrations (up to 250 μM) of the low temperature (average of 36°C) gray sediments that surround the hydrothermal features are low compared to actively venting seafloor. The white patches are warmer (average of 61°C) and feature correspondingly higher pore water H_2_S concentrations (up to 990 μM). The central orange/yellow regions have the highest hydrothermal throughput (average 82°C), but H_2_S concentrations (up to 130 μΜ) appear to be buffered by removal during chemical oxidation to elemental sulfur and amorphous arsenic sulfides [39]. The accumulation of elemental sulfur within the highest temperature regions is consistently observed at the Palaeochori hydrothermal sites. The lack of prominent orange patches at the lower temperature Twinkie site is also consistent with this observation and with previous studies [39],[68],[73],[75],[87]. Temperature differences between these sites are thus a key constraint on the patterns of surficial geochemistry as expressed by seafloor coloration.

Intra-site variation in geochemistry occurs on two different scales; one controlled by the intrinsic heterogeneities of the sediment and another by hydrothermal convection. The film deployments revealed highly dynamic fluid exchange patterns between sulfidic fluids (brown or black stained film) and overlying seawater (gray, unreacted film) (Figure 5). H_2_S exposure on films placed in low temperature sediments with no visible evidence of gas flow (e.g., lack of bubble streams) typically exhibited a gradient of darker staining at the bottom of the film that faded toward the sediment water interface (Figure 5a). These films had a stippled pattern possibly caused by reaction with sulfidic fluids traveling between grain spaces, or the sediment grains themselves may create nucleation points for H_2_S precipitation. In either case, the stippled pattern captures the diffusive transport and mineral grain interactions within low-flow sites. Seafloor ripple marks and the position of the sediment interface are clearly imprinted on these films. In higher temperature white sediments characterized by advective flow, the films were completely darkened (Figure 5b). In one deployment, white filaments bound to the surface of the film, preserving the location of the sediment-water-interface and clearly demonstrating the efflux of H_2_S from the sediments into the overlying bottom waters (note position of white layer in Figure 5b). Regardless of the sediment composition, advective flux completely overwhelmed any localized differences in flow path or mineralogy (e.g., by wave actions, currents, and sediment remobilization). Similar patterns were observed within actively venting sites. Film deployed within a bubble stream reacted quickly (within 30 minutes) (Figure 5c) and retained the pattern of channelized flow from the sediment into the bottom water (Figure 5d). The films captured the flux of H_2_S into the overlying water column at a temporal and spatial resolution that improves upon traditional water sampling methods (pumping or syringe sampling).

Transient fluid flux and sediment heterogeneity are well characterized using the film method. In this study, all H_2_S had a uniform sulfur isotope composition (compare free gas and pore water H_2_S, Figure 9a). Although the H_2_S measured here is isotopically homogenous, exposure patterns suggest the film-capture method is an ideal technique for sampling across chemical and biological gradients.

### Stable isotopes: biogenic vs. abiogenic signatures

The large white patches formed by chemical precipitation of H_2_S-rich and silica-rich hydrothermal fluids at the seafloor host an active microbial community predominated by chemolithotrophic sulfide oxidizing bacteria (e.g., *Thiomicrospira* spp., *Thiobacillus hydrothermalis*, *Achromatium volutans*) and thermophilic sulfate reducing bacteria (e.g., *Desulfacinum spp*) [41],[67]–[70],[72],[73],[87]. Sulfur isotope effects during chemical and biological sulfide oxidation are small (±5‰) [88]–[90] relative to the large isotopic offsets observed during microbial sulfate reduction (up to 66‰) [19],[20]. The process of biological sulfate reduction preferentially produces ^34^S-depleted H_2_S and residual sulfate enriched in ^34^S. Sulfur isotopic fractionation between seawater sulfate and product H_2_S depends on intracellular sulfur transformations during sulfate reduction [91], sulfate reduction rates [20], type of organic substrate [92], microbial community [93], sulfate supply [94], and possibly reoxidation reactions through sulfur disproportionation [95]. Although δ^34^S fractionations between sulfate and H_2_S can be either large (associated with sulfate reduction) or small (associated with sulfide oxidation), δ^18^O fractionations during oxidative and reductive sulfur cycling can both be substantial. Oxygen isotope exchange between intracellular sulfite and water during microbial sulfate reduction produces residual sulfate with high δ^18^O_SO4_[26]. Abiotic sulfide oxidation likewise produces a product sulfate with oxygen that is ^18^O-enriched relative to water or molecular oxygen [27].

Although there is an active community of sulfur oxidizing and reducing bacteria present at the vents, there is no isotope evidence in the bulk geochemical signatures that detects these microbial processes. Microbial sulfate reduction in the sediments would result in a downcore decrease in sulfate concentrations and an associated increase in δ^34^S and δ^18^O of the residual pore water sulfate. No such gradients in pore water sulfate concentration or isotope compositions were present in the upper 20 cm of the sediments. Furthermore, none of the H_2_S extracted from pore water or free gas exhibited the characteristically low δ^34^S_H2S_ values consistent with microbially mediated sulfate reduction (Figure 9). There is also no clear isotopic evidence for sulfur utilization by sulfur-oxidizing bacteria. Previous studies of Milos microbial ecology would suggest that lower temperature white patches would be the most likely areas for an active microbial vent community. Yet, isotope effects (low δ^34^S_SO4_) were only observed within the hottest regions of the vents, not in Twinkie, which is a large white patch that hosts chemolithotrophic bacteria.

The lack of an obvious isotope signature for biotic sulfur cycling within vent and non-vent sediments suggests that *in situ* H_2_S production by microbial sulfate reduction is a minor process relative to the advective (abiotic) H_2_S flux, and that mixing with ambient seawater occurs at a rate sufficient to mask any signal from microbial sulfide oxidation. These results are surprising given that detailed microbial studies indicate the Milos vents are habitat to an active microbial community of sulfate reducers. Genetic sequences (16S rRNA) and abundance data (MPN) demonstrate that thermophilic sulfate reducers of the genus *Desulfacinum* are present within Milos vents [67],[68],[72]. Controlled experiments of natural microbial populations indicate that extant sulfate reducers are well-adapted to low pH and high pCO_2_ conditions of these hydrothermal systems [71]. In that same study, sulfate reduction rate measurements were determined *ex situ* in the laboratory and thus represent the potential rates of microbial sulfate reduction. Based on these experiments, the potential sulfate reduction rates in background sediments were higher than rates achieved in vent sediments [71]. Although the capacity for sulfate reduction is clearly demonstrated, the relative activity of reducers *in situ* may be limited by carbon availability. Previous studies of seagrass beds adjacent to white mats in Palaeochori Bay report high total organic carbon concentrations (0.2 - 3.2%) [73],[87], and sulfate reduction rates (up to 76 μmol SO_4_ dm^−3^ d^−1^) [73] that are similar to those observed at Guaymas Basin and Vulcano Island [33]. In contrast, the vent and non-vent sediments investigated in this study had low organic carbon content (0.04 - 0.08%) and likely low sulfate reduction rates. Furthermore, the films deployed in background sediments showed no visible evidence for reaction with pore water H_2_S.

The relatively low organic carbon content in the sandy sediments of the background and vent sites potentially minimizes biogenic H_2_S generation by microbial sulfate reduction in a setting where abiotic H_2_S appears to predominate. Admittedly, bulk isotope sampling may overlook biological utilization of sulfur within microfabrics or textures at the micron scale. For example, ion microprobe analysis of sulfide minerals (AVS, pyrite, and marcasite) in altered basalt of the West Pacific revealed low δ^34^S characteristic of sulfate reduction and isotopic variability in excess of 30‰ relative to bulk analysis [14]. Such microbial hotspots e.g., [57] are likely present in Palaeochori vents and will be a subject of subsequent studies. Overall, the bulk isotope observations are consistent with carbon and sulfur isotope results reported for the hydrothermally active island of Nisyros. The carbon isotope composition of fumarolic CO_2_ sampled from Nisyros falls on a mixing line between limestone and mid-ocean ridge basalt [96] and the δ^34^S value of free gas H_2_S reflects sulfur derived from a rhyodacite magma [31]. In many locations, Aegean sediments containing organic matter and biogenic H_2_S that would otherwise impart low δ^13^C and low δ^34^S to the subducted lithosphere are thus a minor contribution relative to the flux of abiotic carbon and sulfur sources recycled along the Hellenic Volcanic Arc.

Although biogenic H_2_S contributions are obscured by advection of hydrothermal H_2_S, the sulfur isotope variability observed in sulfate is influenced by hydrothermal input. The majority of pore water δ^34^S_SO4_ were consistent with Palaeochori seawater sulfate (21.2‰; Table 1), but those δ^34^S values that did deviate from normal seawater decreased at higher temperatures (>75°C) (Figure 9). Pore water data show a clear decrease in δ^34^S_SO4_ toward the centers of both Rocky Point and Spiegelei (Figures 10b and c). In contrast, δ^34^S_SO4_ values remain constant in the lower temperature site of Twinkie (Figure 10a). This pattern of low δ^34^S_SO4_ at high temperature suggests that seawater entrained by convective circulation oxidized H_2_S issued from the vents. Sulfide oxidation with molecular oxygen produces a sulfur isotope fractionation of −5.2‰ [88]. Assuming the hydrothermal H_2_S input is large relative to the mass of biogenic H_2_S, chemical oxidation of free gas H_2_S (2.5‰; Table 1) would produce a sulfate (referred herein as secondary sulfate) δ^34^S value of −2.7‰. A two-component mixing model,

(5) $$f_{\mathrm{ss}}=\frac{\delta_{\mathrm{pw}}\;‐\;\delta_{\mathrm{sw}}}{\delta_{\mathrm{gas}}\;‐\;\delta_{\mathrm{sw}}}$$

can then be used to estimate the relative contribution of secondary sulfate (*f*_ss_), assuming the δ^34^S value of sulfate within the pore water (δ_pw_) is a mixture of oxic seawater (δ_sw_ = 21.2‰; Table 1) and sulfate formed from oxidized free gas H_2_S (δ_gas_ = −2.7‰). Isotopic mass balance suggests that approximately 15% (*f*_ss_ = 0.16) of pore water sulfate within the high temperature sites at Spiegelei and Rocky Point is derived from advected H_2_S that was oxidized by seawater entrainment (Figure 11b). If the sulfide oxidation reaction was quantitative (with no attendant fractionation) the secondary sulfate generated by sulfide oxidation could be up to 20% (*f*_ss_ = 0.21). Either estimate demonstrates that a substantial contribution of vent gas-derived H_2_S is incorporated into the local sulfate pool.

The oxygen isotope composition of pore water sulfates in Palaeochori sediments further demonstrates the production of secondary sulfate during seawater entrainment. Residual sulfate δ^34^S and δ^18^O typically evolves toward higher values during microbial sulfate reduction [26]. Contrary to this positive relationship, the paired sulfur and oxygen isotopic composition of sulfates tend to be both ^34^S-depleted and ^18^O-enriched, or invariant δ^34^S coupled with depletion in ^18^O (Figure 12). The departure from Palaeochori seawater sulfate (δ^18^O_SO4_ = 9.0‰) in either a positive or negative direction likely resulted from oxygen isotope exchange during abiotic sulfide oxidation. Spiegelei and Rocky Point pore waters with low pH (<5) and high temperature (>75°C) have high δ^18^O_SO4_ values (Figure 12). Mass balance demonstrates that the low δ^34^S_SO4_ values of these pore waters result from a mixture of seawater sulfate and ^34^S-depleted secondary sulfate produced by sulfide oxidation (Figure 11b). Sulfite, a sulfoxy ion, is an intermediate species produced during both sulfide oxidation and sulfate reduction. Sulfite readily exchanges oxygen with the environment and this equilibrium isotope effect determines the δ^18^O value of sulfate produced by oxidative or reductive sulfur cycling [27]. Ambient sources of oxygen in shallow-sea hydrothermal systems include molecular oxygen (δ^18^O_O2_ = 23.5‰), magmatic water (δ^18^O_H2O_ = 6 to 8‰) and seawater (δ^18^O_H2O_ = −1 to 1.5‰) [24],[97]. It is well demonstrated that seawater altered during high temperature phase separation or water-rock reactions becomes δ^18^O-enriched (by 1 to 2.5‰ at 300°C) [24]. The full extent of oxygen isotope fractionation between newly formed sulfate and available oxygen (Δ^18^O_SO4-H2O_ = 5.9 to 17.6‰) depends on the residence time of sulfite, which rapidly exchanges oxygen at low pH [27]. Regardless of source and the associated isotope effect, the oxygen inherited from acidic and high temperature hydrothermal fluids during abiotic sulfide oxidation is ^18^O-enriched. The high δ^18^O value of geothermal waters on Milos Island (δ^18^O_H2O_ = 4.5‰; aquifer temperature of 330°C) [98] is consistent with this effect.

The oxygen isotope composition of seawater and the hydrothermal fluids were not measured in this study, but the trend toward higher δ^18^O_SO4_ observed in hydrothermal pore waters (Figure 12), is consistent with oxygen isotope exchange via a sulfite intermediate. The isotopic composition of secondary sulfate formed at these sites thus provides a record of both the parent oxygen e.g. [29] and sulfur incorporated during abiotic oxidation.

The secondary sulfate production rates are likely tied to the high spatial and temporal variability of H_2_S delivery from the subsurface. The hydrothermal flux has been shown to fluctuate with tidal pumping, diurnal cycles, and storm activity [47],[65],[68],[69],[73]. In addition, phase-separation (boiling) at these shallow-sea hydrothermal sites can partition seawater into a chloride-rich brine and steam distillate that is low in chloride and enriched in volatile gases such as H_2_S, CO_2_, He, and H_2_[39]. The highly variable thermal regimes resulted in complex pore water chemistry including contributions from a H_2_S-rich gas that may move independently of chloride-rich fluids. The low temperature Twinkie pore waters include phase separated (low chloride) fluids and sulfate concentrations and δ^34^S that were similar to those in seawater. Rocky Point and Spiegelei were higher temperature sites that emit fluids with high chloride concentrations, low sulfate, and δ^34^S_SO4_ that varies with temperature. A second mass balance model normalized to the fractional input of chloride (*f*_brine_) was developed to further constrain the system:

(6) $${\left[{\mathrm{SO}}_{4}^{2‐}\right]}_{\mathrm{pw}}\delta_{\mathrm{pw}}=\;f_{\mathrm{brine}}{\left[{\mathrm{SO}}_{4}^{2‐}\right]}_{\mathrm{brine}}\delta_{\mathrm{brine}}+\left(1‐f_{\mathrm{brine}}\right){\left[{\mathrm{SO}}_{4}^{2‐}\right]}_{\mathrm{sw}}\delta_{\mathrm{sw}}$$

and

(7) $$f_{\mathrm{brine}}=\frac{{\left[{\mathrm{Cl}}^{–}\right]}_{\mathrm{sw}}-{\left[{\mathrm{Cl}}^{–}\right]}_{\mathrm{pw}}}{{\left[{\mathrm{Cl}}^{–}\right]}_{\mathrm{sw}}-{\left[{\mathrm{Cl}}^{–}\right]}_{\mathrm{brine}}}$$

where the sulfate ([SO_4_^2−^]) and chloride ([Cl^−^]) concentrations and δ^34^S values of the pore water sulfate (pw) is a mixture of seawater (sw; δ^34^S = 21.2‰; [SO_4_^2−^] = 32.4 mM; [Cl^−^] = 620.3 mM) and brine. The trajectory of the model array represents the best fit to the observed pore water data (Figure 13). End member values for the brine required to fit the data include secondary sulfate produced by chemical oxidation (δ^34^S_brine_ = −2.7‰), low sulfate concentration ([SO_4_^2−^] = 0.2 mM) and high chloride content ([Cl^−^] = 960 mM). Pore waters from Speigelei and Rocky Point with chloride concentrations in excess of those in Aegean seawater (*f*_brine_ > 0.5) had lower δ^34^S sulfate values (Figure 13). Control samples with negligible fluid inputs (*f*_brine_ = 0) were isotopically identical to seawater.

### Hydrothermal circulation

Downward movement of entraining (cold) oxic seawater and buoyant upward flow of (hot) fluids establish convective circulation in which solutions pass through multiple reactions zones during transport in the subsurface [4]. Regardless of the chemical pathway, an equilibrium isotope effect between dissolved H_2_S and anhydrite (CaSO_4_) veins precipitated near the seafloor can buffer the δ^34^S of evolved fluids [6]. Anhydrite is a common hydrothermal mineral that forms during retrograde solubility of seawater sulfate at temperatures above 150°C [99],[100]. H_2_S in the ascending fluids will equilibrate with sulfate in the anhydrite front, and the extent of equilibration depends upon temperature and residence time of the fluid that comes into contact with the anhydrite. Multiple sulfur isotope (^32^S, ^33^S, ^34^S) mass balance models indicate that the anhydrite buffer model imparts a final filter on the isotope signature of fluids that discharge on the seafloor. Based on these isotope models, a significant portion of vent sulfide in the Mid-Atlantic Ridge and East Pacific Rise is derived from seawater sulfate (22% to 33%) [12],[13].

**Figure 14:**
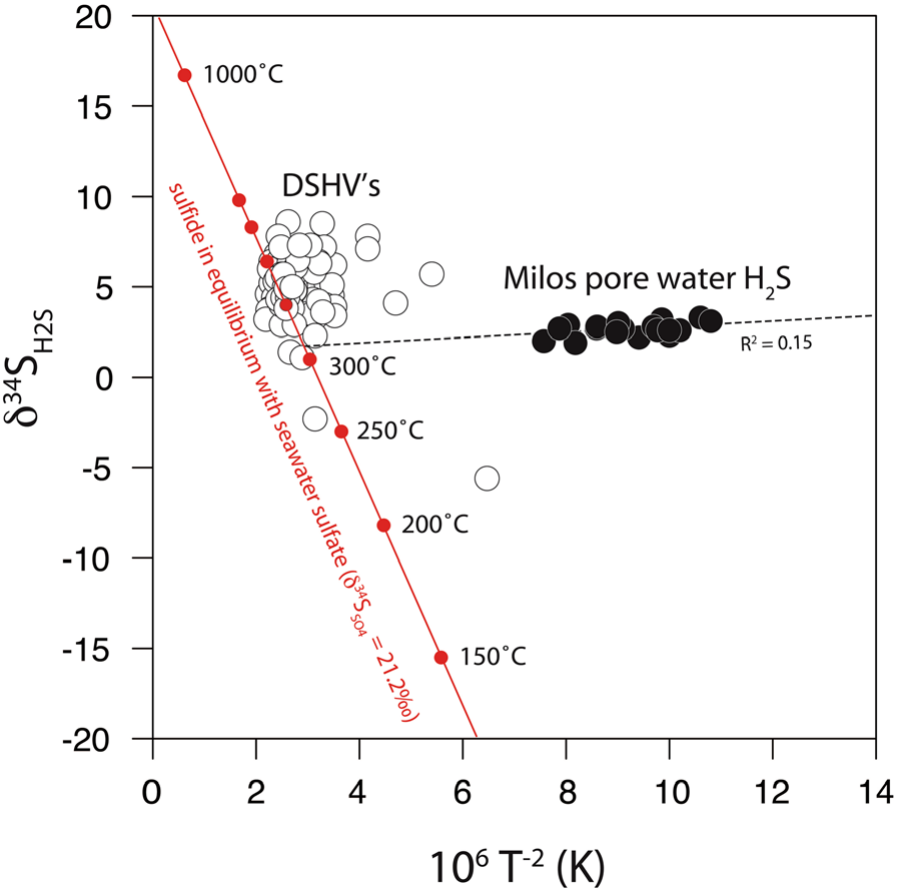
**Temperature and δ**^**34**^**S of hydrothermal sulfide from deep-sea hydrothermal vents (DSHV’s; open symbols) and Milos pore waters (solid symbols).** Estimates calculated for sulfide in equilibrium (red line) with seawater sulfate (δ^34^S_SO4_ = 21.2‰). The Milos regression intersects the equilibrium model at 311.4°C and δ^34^S_H2S_ = 1.7‰.

In this study of the upper 20 cm of the Palaeochori seafloor sediment, δ^34^S and temperature data are consistent with partial isotopic exchange between vent H_2_S and subsurface anhydrite (Figure 14). Isotopic exchange between sulfate and dissolved H_2_S increases with temperature according to the empirical equilibrium model:

(8) $$1000\ln\alpha \;=\;\frac{6.463\;\times \;10^{6}}{T^{2}}\;+\;0.56\;\left(\pm 0.5\right)$$

where the fractionation factor between sulfate and H_2_S (α) is inversely proportional to temperature (T, in Kelvin) [101]. H_2_S in exchange with anhydrite approaches seawater values (δ^34^S_SO4_ = 21.2‰) at temperatures above 1273.2 K (1000°C). Deep-sea hydrothermal vent H_2_S (>1500 m water depth) have δ^34^S values that approximate high temperature equilibrium exchange with seawater sulfate (Figure 14). In contrast, Palaeochori H_2_S is out of isotopic equilibrium with seawater sulfate (δ^34^S_SO4_ = 21.2‰), yet these data fall along a mixing line that intercepts the equilibrium line at a buffered H_2_S value of 1.7‰ and 311.4°C. The δ^34^S values track a temperature dependent array from this initial value up to a maximum δ^34^S of 3.3‰ in the lower temperature background sediments (33.5°C). This linear departure from the initial δ^34^S value could represent an array of isotopic signatures attained at high temperature and those altered during non-equilibrium (enzymatic) reactions, such as microbial sulfate reduction in the low temperature sediments. Inorganic disproportionation of magmatic SO_2_ is another potential isotope fractionation mechanism that can produce ^34^S-enriched sulfate (by 16 to 21‰) and a residual H_2_S with low δ^34^S [28]; however, SO_2_ has not been detected in Milos vents e.g. [41] and vent H_2_S is not exceptionally depleted in ^34^S.

The ~300°C temperature estimate is consistent with geothermometry calculations for the deep-seated hydrothermal reservoir. Reaction temperatures estimated from phase equilibrium Na-K-Ca geothermometry of volcanic fluids from Milos suggests a 300-325°C reservoir [69],[102] positioned at 1–2 km depth and a shallow 248°C reservoir at 0.2-0.4 km [102]. Similar deep reservoir temperatures (345°C) and a phase separation temperature (260°C) were estimated from gas geothermometry (H_2_-Ar, H_2_-N_2_, H_2_-H_2_O) at Nysiros [96].

## Conclusions

Much of the current understanding of hydrothermal cycling of sulfur and carbon is based on major element and isotope systematics developed from investigations of altered basalts in trenches and new crust formed along spreading centers. In general, sulfur contributions to submarine hydrothermal vents are derived from sulfur mobilized from host rock and seawater sulfate reduced during thermochemical or microbial sulfate reduction. The felsic to intermediate composition of magma at Milos and other shallow-sea vents results in vent fluids with wide-ranging chemistries. The shallow depths also expose these igneous fluids to physical mixing (tidal or wind-driven), phase separation, and microbial utilization. Chemical and biological reactions in these systems are dynamic over small spatial scales and short temporal scales. Shallow-sea hydrothermal vents along continental margins and convergence zones such as Milos have geochemical and environmental conditions that are unique from deep-sea counterparts.

The Milos vents are characterized by white (lower temperature) and orange/yellow (higher temperature) seafloor precipitates. Sulfide-sensitive films deployed in colored seafloor and background sediments captured the diffusive or advective nature of fluid discharge. Pore fluids analyzed from these same sites revealed a highly uniform sulfur isotope value for H_2_S in the vent gases and pore waters (δ^34^S_H2S_ = 2.5‰). The shifts toward low δ^34^S_H2S_, and high δ^34^S_SO4_ and δ^18^O_SO4_ characteristic of microbial sulfate reduction was not observed within any of the sites. Sulfur isotope evidence does suggest that pore fluids in high temperature sites contain a mixture of entrained oxic seawater and a ^34^S-depleted pool of secondary sulfate. An equilibrium isotope model suggests that volcanic inputs are buffered to an initial δ^34^S_H2S_ value of 1.7‰ by subsurface anhydrite veins. At these shallow-sea hydrothermal vent sites, the normally diagnostic biosignatures of microbial sulfate reduction (low δ^34^S_H2S_ and high δ^34^S_SO4_ and δ^18^O_SO4_) were not readily differentiated from igneous sulfur inputs. Improved knowledge obtained here about the interactions between the biotic and abiotic sulfur cycle within complex natural environments will further refine geochemical proxies for biologically mediated processes recorded in the geologic record.

## Competing interests

The authors declare that they have no competing interests.

## Authors’ contributions

DF, GD, JA, and WG conceived of the study, and participated in its design and coordination. DF, GD, JA, RP, and WG conducted the fieldwork and sample collection. WG prepared and analyzed samples for isotopic analysis. RP and JA measured pH and temperature *in situ*. RP analyzed anion concentrations. GD conducted the voltammetry and FK calibrated the electrodes for the accurate determination of dissolved H_2_S concentrations. WG, DF, and GD drafted the manuscript. RP and JA provided assistance editing and finalizing the manuscript. All authors read and approved the final manuscript.

## References

[B1] Clark BC Billingham J Sulfur: Fountainhead of Life in the Universe Life in the Universe 1981 MIT Press, Cambridge 47 60

[B2] Jørgensen BB Mineralization of organic matter in the sea bed - the role of sulphate reduction Nature 1982 296 643 645

[B3] Berner RA Burial of organic carbon and pyrite sulfur in the modern ocean: its geochemical and environmental significance Am J Sci 1982 282 451 473

[B4] Gamo T Sakai H, Nozaki Y Wide variation of chemical characteristics of submarine hydrothermal fluids due to secondary modification processes after high temperature water-rock interaction: a review Biogeochemical Processes and Ocean Flux in the Western Pacific 1995 Terra Scientific, Tokyo 425 451

[B5] Henley RW Ellis AJ Geothermal Systems Ancient and Modern: A Geochemical Review Earth Sci Rev 1983 19 1 50

[B6] Ohmoto H Goldhaber MB Barnes HL Sulfur and Carbon Isotopes Geochemistry of Hydrothermal Ore Deposits 1997 John Wiley & Sons, New York 517 611

[B7] Butterfield DA Jonassan IR Massoth GJ Feely RA Roe KK Embley RE Holden JF McDuff RE Lilley MD Delaney JR Seafloor eruptions and evolution of hydrothermal fluid chemistry Philos Trans R Soc A 1997 355 369 386

[B8] Elderfield H Schultz A Mid-ocean Ridge hydrothermal fluxes and the chemical composition of the ocean Annu Rev Earth Planet Sci 1996 24 191 224

[B9] Staudigel H Hart SR Alteration of basaltic glass: Mechanisms and significance for the oceanic crust-seawater budget Geochim Cosmochim Acta 1983 47 337 350

[B10] Edmond JM Measures C McDuff RE Chan LH Collier R Grant B Gordon LI Corliss JB Ridge crest hydrothermal activity and the balances of the major and minor elements in the ocean: The Galapagos data Earth Planet Sci Lett 1979 46 1 18

[B11] German CR Von Damm KL Holland HD, Turekian KK Hydrothermal Processes Treatise on Geochemistry 2003 Elsevier, Oxford 181 222

[B12] Ono S Shanks WC III Rouxel OJ Rumble D S-33 contraints on seawater sulfate contribution in modern seafloor hydrothermal vent sulfides Geochim Cosmochim Acta 2007 71 1170 1182

[B13] Peters M Strauss H Farquhar J Ockert C Eickmann B Jost CL Sulfur cycling at the Mid-Atlantic Ridge: A multiple sulfur isotope approach Chem Geol 2010 269 180 196

[B14] Rouxel O Ono S Alt J Rumble D Ludden J Sulfur isotope evidence for microbial sulfate reduction in altered oceanic basalts at ODP Site 801 Earth Planet Sci Lett 2008 268 110 123

[B15] Sakai H Des Marais DJ Ueda A Moore JG Concentrations and isotope ratios of carbon, nitrogen and sulfur in ocean-floor basalts Geochim Cosmochim Acta 1984 48 2433 2441 11540821 10.1016/0016-7037(84)90295-3

[B16] Rees CE Jenkins WJ Monster J The sulphur isotopic composition of ocean water sulphate Geochim Cosmochim Acta 1978 42 377 381

[B17] Chaussidon M Albarede F Sheppard SMF Sulphur isotope variations in the mantle from ion microprobe analyses of micro-sulphide inclusions Earth Planet Sci Lett 1989 92 144 156

[B18] Chaussidon M Albarede F Sheppard SMF Sulphur isotope heterogeneity in the mantle from ion microprobe measurements of sulphide inclusions in diamonds Nature 1987 330 242 244

[B19] Canfield DE Valley JW, Cole DR Biogeochemistry of Sulfur Isotopes Stable Isotope Geochemistry 2001 Mineralogical Society of America, Washington DC 607 636

[B20] Sim MS Ono S Donovan K Templer SP Bosak T Effect of electron donors on the fractionation of sulfur isotopes by a marine *Desulfovibrio* sp Geochim Cosmochim Acta 2011 75 4244 4259

[B21] Alt JC Burdett JW Larson RL, Lancelot Y Sulfur in Pacific deep-sea sediments (Leg 129) and implications for cycling of sediment in subduction zones Proc. ODP, Sci. Results, 129: College Station TX (Ocean Drilling Program) 1992 283 294

[B22] Canfield DE The Evolution of the Earth Surface Sulfur Reservoir Am J Sci 2004 304 839 861

[B23] Shanks WC Stable Isotopes in Seafloor Hydrothermal Systems: Vent fluids, hydrothermal deposits, hydrothermal alteration, and microbial processes Rev Mineral Geochem 2001 43 469 525

[B24] Shanks WC III Böhlke JK Seal RR II Stable isotopes in mid-ocean ridge hydrothermal systems: Interactions between fluids, minerals, and organisms Seafloor Hydrothermal Systems: Physical, Chemical, Biological, and Geological Interactions 1995 AGU, Washington, DC 194 221

[B25] Chiba H Sakai H Oxygen isotope exchange rate between dissolved sulfate and water at hydrothermal temperatures Geochim Cosmochim Acta 1985 49 993 1000

[B26] Brunner B Bernasconi SM Kleikemper J Schroth MH A model for oxygen and sulfur isotope fractionation in sulfate during bacterial sulfate reduction processes Geochim Cosmochim Acta 2005 69 4773 4785

[B27] Müller IA Brunner B Coleman M Isotopic evidence of the pivotal role of sulfite oxidation in shaping the oxygen isotope signature of sulfate Chem Geol 2013 354 186 202

[B28] Craddock PR Bach W Insights to magmatic-hydrothermal processes in Manus back-arc basin as recorded by anhydrite Geochim Cosmochim Acta 2010 74 5514 5536

[B29] Teagle DAH Alt JC Halliday AN Tracing the chemical evolution of fluids during hydrothermal recharge: Constraints from anhydrite recovered in ODP Hole 504B Earth Planet Sci Lett 1998 155 167 182

[B30] Marini L Fiebig J Hunziker JC, Marini L Fluid geochemistry of the magmatic-hydrothermal system of Nisyros (Greece) The Geology, Geochemistry and Evolution of Nisyros Volcano (Greece). Implications for the Volcanic Hazards 2005 Section des sciences de la Terre, Université de Lausanne, Lausanne 121 163

[B31] Marini L Gambardella B Principe C Arias A Brombach T Hunziker JC Characterization of magmatic sulfur in the Aegean island arc by means of the δ^34^S values of fumarolic H_2_S, elemental S, and hydrothermal gypsum from Nisyros and Milos Islands Earth Planet Sci Lett 2002 200 15 31

[B32] Peters M Strauss H Petersen S Kummer N Thomazo C Hydrothermalism in the Tyrrhenian Sea: Inorganic and microbial sulfur cycling as revealed by geochemical and multiple sulfur isotope data Chem Geol 2011 280 217 231

[B33] Amend JP Rogers KL Meyer-Dombard DR Amend JP, Edwards KJ, Lyons TW Microbially mediate sulfur-redox: Energetics in marine hydrothermal vent systems Sulfur Biogeochemistry - Past and Present: Geological Society of America Special Paper 379 2004 Geological Society of America, Boulder 17 34

[B34] Tarasov VG Gebruk AV Mironov AN Moskalev LI Deep-sea and shallow-water hydrothermal vent communities: Two different phenomena? Chem Geol 2005 224 5 39

[B35] Butterfield DA Massoth GJ McDuff RE Lupton JE Lilley MD Geochemistry of hydrothermal fluids from Axial Seamount hydrothermal emissions study vent field, Juan de Fuca Ridge: Subseafloor boiling and subsequent fluid-rock interaction J Geophys Res 1990 95 12895 12922

[B36] Bischoff JL Rosenbauer RJ The critical point and two-phase boundary of seawater, 200–500°C Earth Planet Sci Lett 1984 68 172 180

[B37] Foustoukos DI Seyfried WE Fluid Phase Separation Processes in Submarine Hydrothermal Systems Rev Mineral Geochem 2007 65 213 239

[B38] Ishibashi J-i Urabe T Taylor B Hydrothermal Activity Related to Arc-Backarc Magmatism in the Western Pacific Backarc Basins 1995 Springer US, New York 451 495

[B39] Price RE Savov I Planer-Friedrich B Bühring SI Amend JP Pichler T Processes influencing extreme As enrichment in shallow-sea hydrothermal fluids of Milos Island, Greece Chem Geol 2013 348 15 26

[B40] Druschel GK Rosenberg PE Non-magmatic fracture-controlled hydrothermal systems in the Idaho Batholith: South Fork Payette geothermal system Chem Geol 2001 173 271 291

[B41] Dando PR Aliani S Arab H Bianchi CN Brehmer M Cocito S Fowler SW Gundersen J Hooper LE Kölbl R Kuever J Linke P Makropoulos KC Meloni R Miquel J-C Morri C Müller S Robinson C Schlesner H Sievert SM Stöhr R Stüben D Thomm M Varnavas SP Ziebis W Hydrothermal Studies in the Aegean Sea Physics and Chemistry of the Earth, Part B: Hydrology, Oceans and Atmosphere 2000 25 1 8

[B42] Dando PR Hughes JA Leahy Y Niven SJ Taylor LJ Smith C Gas venting rates from submarine hydrothermal areas around the island of Milos, Hellenic Volcanic Arc Continent Shelf Res 1995 15 913 929

[B43] Price RE Amend JP Pichler T Enhanced geochemical gradients in a marine shallow-water hydrothermal system: Unusual arsenic speciation in horizontal and vertical pore water profiles Appl Geochem 2007 22 2595 2605

[B44] Price RE Pichler T Distribution, speciation and bioavailability of arsenic in a shallow-water submarine hydrothermal system, Tutum Bay, Ambitle Island, PNG Chem Geol 2005 224 122 135

[B45] Sansone FJ Pawlak G Stanton TP McManus MA Glazer BT DeCarlo EH Bandet M Sevadjian J Stierhoff K Colgrove C Hebert AB Chen IC Kilo Nalu: Physical/biogeochemical dynamics above and within permeable sediments Oceanography 2008 21 173 178

[B46] Hebert AB Sansone FJ Pawlak GR Tracer dispersal in sandy sediment porewater under enhanced physical forcing Continent Shelf Res 2007 27 2278 2287

[B47] Yücel M Sievert SM Vetriani C Foustoukos DI Giovannelli D Le Bris N Eco-geochemical dynamics of a shallow-water hydrothermal vent system at Milos Island, Aegean Sea (Eastern Mediterranean) Chem Geol 2013 356 11 20

[B48] Truesdell A Nehring N Gases and water isotopes in a geochemical section across the Larderello, Italy, geothermal field Pure Appl Geophys 1978 117 276 289

[B49] Pichler T Veizer J Hall GEM The chemical composition of shallow-water hydrothermal fields in Tutum Bay, Ambitle Island, Papua New Guinea and their effect on ambient seawater Mar Chem 1999 64 229 252

[B50] Luther GW III The role of one and two electron transfer reactions in forming thermodynamically unstable intermediates as barriers in multi-electron redox reactions Aquat Geochem 2010 16 395 420

[B51] Zopfi J Ferdelman TG Fossing H Amend JP, Edwards KJ, Lyons TW Distribution and fate of sulfur intermediates - sulfide, tetrathionate, thiosulfate, and elemental sulfur - in marine sediments Sulfur Biogeochemistry - Past and Present: Geological Society of America Special Paper 379 2004 Geological Society of America, Boulder 97 116

[B52] Werne JP Hollander DJ Lyons TW Sinninghe Damste JS Amend JP, Edwards KJ, Lyons TW Organic sulfur biogeochemistry: Recent advances and furture research directions Sulfur Biogeochemistry - Past and Present: Geological Society of America Special Paper 379 2004 Geological Society of America, Boulder 135 150

[B53] Gartman A Yücel M Madison AS Chu DW Ma S Janzen CP Becker EL Beinart RA Girguis PR Luther GW III Sulfide Oxidation across Diffuse Flow Zones of Hydrothermal Vents Aquat Geochem 2011 17 583 601

[B54] Millero FJ The thermodynamics and kinetics of the hydrogen sulfide system in natural waters Mar Chem 1986 18 121 147

[B55] Millero FJ Estimate of the life time of superoxide in seawater Geochim Cosmochim Acta 1987 51 351 353

[B56] Luther GW Findlay AJ MacDonald DJ Owings SM Hanson TE Beinart RA Girguis PR Thermodynamics and kinetics of sulfide oxidation by oxygen: A look at inorganically controlled reactions and biologically mediated processes in the environment Front Microbiol 2011 2 1 9 21833317 10.3389/fmicb.2011.00062 PMC3153037

[B57] Fike DA Gammon CL Ziebis W Orphan VJ Micron-scale mapping of sulfur cycling across the oxycline of a cyanobacterial mat: a paired nanoSIMS and CARD-FISH approach ISME J 2008 2 749 759 18528418 10.1038/ismej.2008.39

[B58] Fike DA, Finke N, Zha J, Blake G, Hoehler TM, Orphan VJ: **The effect of sulfate concentration on (sub)millimeter-scale sulfide δ**^ **34** ^**S in hypersaline cyanobacterial mats over the diel cycle.** *Geochim Cosmochim Acta* 2009, **73:**6187–6204.

[B59] Berner RA Raiswell R Burial of organic carbon and pyrite sulfur in sediments over Phanerozoic time: a new theory Geochim Cosmochim Acta 1983 47 855 862

[B60] Kump LR Garrels RM Modelling atmospheric O_2_ in the global sedimentary redox cycle Am J Sci 1986 286 337 360

[B61] Gaillard F Scaillet B Arndt NT Atmospheric oxygenation caused by a change in volcanic degassing pressure Nature 2011 478 229 233 21993759 10.1038/nature10460

[B62] Kasting JF Catling DC Zahnle K Atmospheric oxygenation and volcanism Nature 2012 487 E1 E2 22837006 10.1038/nature11274

[B63] Fytikas M Updating of the goelogical and geothermal research on Milos Island Geothermics 1989 18 485 496

[B64] Kilias SP Nomikou P Papanikolaou D Polymenakou PN Godelitsas A Argyraki A Carey S Gamaletsos P Mertzimekis TJ Stathopoulou E Goettlicher J Steininger R Betzelou K Livanos I Christakis C Bell KC Scoullos M New insights into hydrothermal vent processes in the unique shallow-submarine arc-volcano, Kolumbo (Santorini), Greece Sci Rep 2013 3 1 13 10.1038/srep02421 PMC3741630 23939372

[B65] Varnavas SP Cronan DS Submarine hydrothermal activity off Santorini and Milos in the Central Hellenic Volcanic Arc: A synthesis Chem Geol 2005 224 40 54

[B66] Valsami-Jones E Baltatzis E Bailey EH Boyce AJ Alexander JL Magganas A Anderson L Waldron S Ragnarsdottir KV The geochemistry of fluids from an active shallow submarine hydrothermal system: Milos Island, Hellenic Volcanic Arc J Volcanol Geoth Res 2005 148 130 151

[B67] Sievert SM Kuever J Desulfacinum hydrothermale sp. nov., a thermophilic, sulfate-reducing bacterium from geothermally heated sediments near Milos Island (Greece) Int J Syst Evol Microbiol 2000 50 1239 1246 10843068 10.1099/00207713-50-3-1239

[B68] Sievert SM Brinkhoff T Muyzer G Ziebis W Kuever J Spatial Heterogeneity of Bacterial Populations along an Environmental Gradient at a Shallow Submarine Hydrothermal Vent near Milos Island (Greece) Appl Environ Microbiol 1999 65 3834 3842 10473383 10.1128/aem.65.9.3834-3842.1999 PMC99708

[B69] Fitzsimons MF Dando PR Hughes JA Thiermann F Akoumainaki I Pratt SM Submarine hydrothermal brine seeps off Milos, Greece: Observations and geochemistry Mar Chem 1997 57 325 340

[B70] Brinkhoff T Sievert SM Kuever J Muyzer G Distribution and Diversity of Sulfur-Oxidizing *Thiomicrospira* spp. at a Shallow-Water Hydrothermal Vent in the Aegean Sea (Milos, Greece) Appl Environ Microbiol 1999 65 3843 3849 10473384 10.1128/aem.65.9.3843-3849.1999 PMC99709

[B71] Bayraktarov E Price RE Ferdelman TG Finster K The pH and pCO_2_ dependence of sulfate reduction in shallow-sea hydrothermal CO_2_-venting sediments (Milos Island, Greece) Front Microbiol 2013 4 1 10 23658555 10.3389/fmicb.2013.00111 PMC3647119

[B72] Price RE Lesniewski R Nitzsche K Meyerdierks A Saltikov C Pichler T Amend J Archaeal and bacterial diversity in an arsenic-rich shallow-sea hydrothermal system undergoing phase separation Front Microbiol 2013 4 1 19 23847597 10.3389/fmicb.2013.00158 PMC3705188

[B73] Dando PR Hughes JA Thiermann F Parson LM, Walker CL, Dixon DR Preliminary observations on biological communities at shallow hydrothermal vents in the Aegean Sea Hydrothermal Vents and Processes, Geological Society Special Publication No. 87 1995 303 317

[B74] Naden J Kilias SP Darbyshire DPF Active geothermal systems with entrained seawater as modern analogs for transitional volcanic-hosted massive sulfide and continental magmato-hydrothermal mineralization: The example of Milos Island, Greece Geology 2005 33 541 544

[B75] Wenzhöfer F Holby O Glud RN Nielsen HK Gundersen JK In situ microsensor studies of a shallow water hydrothermal vent at Milos, Greece Mar Chem 2000 69 43 54

[B76] Brendel PJ Luther GW Development of a gold amalgam voltammetric microelectrode for the determination of dissolved Fe, Mn, O2, and S(−II) in porewaters of marine and freshwater sediments Environ Sci Technol 1995 29 751 761 22200285 10.1021/es00003a024

[B77] Druschel G Baker B Gihring T Banfield J Acid mine drainage biogeochemistry at Iron Mountain, California Geochem Trans 2004 5 1 20 10.1186/1467-4866-5-13 PMC1475782 35412773

[B78] Luther GW III Glazer BT Ma S Trowborst RE Moore TS Metzger E Kraiya C Waite TJ Druschel G Sundby B Taillefert M Nuzzio DB Shank TM Lewis B Brendel PJ Use of voltammetric solid-state (micro)electrodes for studying biogeochemical processes: Laboratory measurements to real time measurements with an in situ electrochemical analyzer (ISEA) Mar Chem 2008 108 221 235

[B79] Taillefert M Rozan TF Taillefert M, Rozan TF Electrochemical methods for the environmental analysis of trace elements biogeochemistry Environmental Electrochemistry: Analyses of Trace Element Biogeochemistry 2002 American Chemical Society, Washington DC 3 14

[B80] Druschel GK Hamers RJ Luther GW Banfield JF Kinetics and mechanism of trithionate and tetrathionate oxidation at low pH by hydroxyl radicals Aquat Geochem 2003 9 145 164

[B81] Luther GW III Glazer BT Hohmann L Popp J Taillefert M Rozan TF Brendel PJ Therberge SM Nuzzio DB Sulfur speciation monitored in situ with solid state gold amalgam voltammetric microelectrodes: polysulfides as a special case in sediments, microbial mats and hydrothermal vent waters J Environ Monit 2001 3 61 66 11253020 10.1039/b006499h

[B82] Meites L Handbook of Analytical Chemistry 1961 McGraw-Hill, New York

[B83] Slowey AJ DiPasquale MM How to overcome inter-electrode variability and instability to quantify dissolved oxygen, Fe(II), Mn(II), and S(−II) in undisturbed soils and sediments using voltammetry Geochem Trans 2012 13 1 20 22731822 10.1186/1467-4866-13-6 PMC3442984

[B84] Canfield DE Raiswell R Westrich JT Reaves CM Berner RA The use of chromium reduction in the analysis of reduced inorganic sulfur in sediments and shales Chem Geol 1986 54 149 155

[B85] Kamyshny A Jr Goifman A Gun J Rizkov D Lev O Equilibrium Distribution of Polysulfide Ions in Aqueous Solutions at 25°C: a new approach for the study of polysulfides’ equilibria Environ Sci Tech 2004 38 6633 6644 10.1021/es049514e 15669322

[B86] Karageorgis A Anagnostou C Sioulas A Chronis G Papathanassiou E Sediment geochemistry and mineralogy in Milos bay, SW Kyklades, Aegean Sea, Greece J Mar Syst 1998 16 269 281

[B87] Thiermann F Akoumainaki I Hughes JA Giere O Benthic fauna of a shallow-water gaseohydrothermal vent area in the Aegean Sea (Milos, Greece) Mar Biol 1997 128 149 159

[B88] Fry B Ruf W Gest H Hayes JM Sulfur Isotope Effects Associated with Oxidation of Sulfide by O_2_ in Aqueous Solution Chem Geol 1988 73 205 210 10.1016/0168-9622(88)90001-2 11538336

[B89] Fry B Gest H Hayes JM Isotope effects associated with the anaerobic oxidation of sulfide by the purple photosynthetic bacterium, Chromatium vinosum FEMS Microbiol Lett 1984 22 283 287 10.1016/0378-1097(85)90318-0 11540842

[B90] Zerkle AL Farquhar J Johnston DT Cox RP Canfield DE Fractionation of multiple sulfur isotopes during phototrophic oxidation of sulfide and elemental sulfur by a green sulfur bacterium Geochim Cosmochim Acta 2009 73 291 306

[B91] Bradley AS Leavitt WD Johnston DT Revisiting the dissimilatory sulfate reduction pathway Geobiology 2011 9 446 457 21884365 10.1111/j.1472-4669.2011.00292.x

[B92] Detmers J Bruchert V Habicht KS Kuever J Diversity of Sulfur Isotope Fractionations by Sulfate-Reducing Prokaryotes Appl Environ Microbiol 2001 67 888 894 11157259 10.1128/AEM.67.2.888-894.2001 PMC92663

[B93] Brüchert V Knoblauch C Jørgensen BB Controls on stable sulfur isotope fractionation during bacterial sulfate reduction in Arctic sediments Geochim Cosmochim Acta 2001 65 763 776

[B94] Chanton J Martens C Goldhaber M Biogeochemical cycling in an organic-rich coastal marine basin. 8. A sulfur isotopic budget balanced by differential diffusion across the sediment-water interface Geochim Cosmochim Acta 1987 51 1201 1208

[B95] Habicht KS Canfield DE Rethmeier J Sulfur isotope fractionation during bacterial reduction and disproportionation of thiosulfate and sulfite Geochim Cosmochim Acta 1998 62 2585 2595

[B96] Brombach T Caliro S Chiodini G Fiebig J Hunziker JC Raco B Geochemical evidence for mixing of magmatic fluids with seawater, Nisyros hydrothermal system, Greece Bull Volcanol 2003 65 505 516

[B97] Kroopnick P Craig H Atmospheric Oxygen: Isotopic Composition and Solubility Fractionation Science 1972 175 54 55 17833979 10.1126/science.175.4017.54

[B98] Dotsika E Poutoukis D Michelot JL Raco B Natural tracers for indentifying the origin of the thermal fluids emerging along the Aegean Volcanic arc (Greece): Evidence of Arc-Type Magmatic Water (ATMW) participation J Volcanol Geoth Res 2009 179 19 31

[B99] Shanks WC III Bischoff JL Rosenbauer RJ Seawater sulfate reduction and sulfur isotope fractionation in basaltic systems: Interaction of seawater with fayalite and magnetite at 200–350°C Geochim Cosmochim Acta 1981 45 1977 1995

[B100] Sleep NH Hydrothermal circulation, anhydrite precipitation, and thermal structure at ridge axes J Geophys Res 1991 96 2375 2387

[B101] Ohmoto H Lasaga AC Kinetics of reactions between aqueous sulfates and sulfides in hydrothermal systems Geochim Cosmochim Acta 1982 46 1727 1745

[B102] Wu S You C Wang B Valsami-Jones E Baltatzis E Two-cells phase separation in shallow submarine hydrothermal system at Milos Island, Greece: Boron isotopic evidence Geophys Res Lett 2011 38 L08613

